# Tailoring the Melting and Glass Transition Behavior of Zeolitic Imidazolate Frameworks via Ammonium Halide Salts

**DOI:** 10.1002/smll.73675

**Published:** 2026-05-08

**Authors:** Fengming Cao, Søren S. Sørensen, Anders K. R. Christensen, Xuan Ge, Martin A. Karlsen, Giulio Monaco, Lothar Wondraczek, Morten M. Smedskjaer

**Affiliations:** ^1^ Department of Chemistry and Bioscience Aalborg University Aalborg Denmark; ^2^ Shanghai Key Laboratory of Materials Laser Processing and Modification School of Materials Science and Engineering Shanghai Jiao Tong University Shanghai PR China; ^3^ Deutsches Elektronen‐Synchrotron (DESY) Hamburg Germany; ^4^ Department of Physics and Astronomy `Galileo Galilei' University of Padova Padova Italy; ^5^ Otto Schott Institute of Materials Research Friedrich Schiller University Jena Jena Germany

## Abstract

Metal‐organic framework (MOF) glasses represent a promising class of organic‐inorganic hybrid materials, but most crystalline MOFs undergo thermal decomposition before melting and can thus not be quenched into a glassy state. To this end, recent work has introduced the concept of MOF modifiers, i.e., the mixing of a modifier agent with a MOF crystal to lower the MOF's melting temperature. While previous work has focused on hybrid modifiers, we here investigate the use of inorganic ammonium halide salts as modifiers for zeolitic imidazolate frameworks (ZIFs). We show that co‐melting various ZIF crystals with the halide salt significantly lowers both melting and glass transition temperatures, enabling fabrication of centimeter‐sized hybrid glasses and melt‐processing of ZIF‐8, which otherwise decomposes before melting. In situ X‑ray imaging is used to visualize that the molten halide salts infiltrate the ZIF crystals and ultimately promote the collapse of the crystalline domains into a homogeneous melt. X‐ray pair distribution function analyses confirm the disruption of the Zn─N coordination network and formation of Zn─X (X = Cl, Br, and I) bonding, thus lowering the framework connectivity. Our findings broaden the scope of modified MOF glasses and offer a versatile route for tailoring their thermal processing and network structure.

## Graphical abstract

Ammonium halide salts serve as inorganic modifiers that lower the melting and glass transition temperatures of zeolitic imidazolate frameworks, enabling centimeter‐scale hybrid glasses and melt processing of otherwise unmeltable ZIF‐8. In situ imaging and pair distribution function analysis reveal salt infiltration, Zn─N bond disruption, and Zn–halide bond formation that reconfigure framework connectivity.

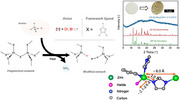

## 1 Introduction

Metal‐organic frameworks (MOFs) are a class of crystalline porous materials constructed from metal nodes and organic linkers through coordination bonds [1, 2]. They have garnered significant attention in recent years due to their outstanding properties, such as high surface areas, tunable pore structures, and chemical versatility [3]. These form the basis for a wide range of potential applications, including gas storage and separation, catalysis, sensing, and drug delivery [2, 4, 5, 6, 7]. However, most of the ∼10^5^ MOF crystals discovered so far are practically only accessible in the form of polycrystalline powders [8, 9], although it is possible to fabricate MOF thin films [10, 11] and mm‐sized single crystals [12]. This limits their processability and ability to form large, homogeneous, and mechanically stable bulk geometries. For example, they can instead be used as a coating, but this may generate other problems, such as pore blocking and lower amounts of active material [8, 13]. To enable more applications, it would be beneficial for MOF structures to be mechanically self‐supporting and free of grain boundaries.

The development of meltable MOFs and therefrom MOF glasses offers a promising route to address this limitation [14, 15]. This method enables the formation of bulk glassy materials while retaining the short‐range chemical connectivity of the original framework, thus offering unique opportunities for structural and functional tuning beyond the crystalline state [16, 17, 18]. So far, this has allowed the formation of cm‐sized MOF glasses with some degree of retained porosity [19, 20]. However, only a limited number of MOFs can undergo melt‐quenching due to thermal decomposition prior to melting. Among these, zeolitic imidazolate frameworks (ZIFs) represent a notable subclass capable of forming glasses, such as ZIF‐62 ([Zn(Im)_1.75_(bIm)_0.25_], where Im is imidazolate and bIm is benzimidazolate) [21, 22, 23], ZIF‐4 ([Zn(Im)_2_]) [24, 25], ZIF‐76 (Zn(Im)_1.62_(5‐ClbIm)_0.38_, where 5‐ClbIm is 5‐chlorobenzimidazolate) [26] and TIF‐4 ([Zn(Im)_1.5_(mbIm)_0.5_], where 5‐mbIm is 5‐methylbenzimidazole) [24]. However, other ZIFs, such as the highly porous and extensively studied ZIF‐8 (Zn(mIm)_2_, where mIm is 2‐methylimidazole) [27], undergo thermal decomposition before melting. This limitation underscores the need for more generalizable strategies to enable melt‐quenching and glass formation across a broader range of MOFs.

To expand the number of meltable MOFs, the temperature required for melting and glass formation could be lowered. Various strategies have been adopted to enable this, including melting at elevated pressure [28, 29], exchange of organic ligands (linkers) [30], and incorporation of ionic liquids [8, 31]. As an alternative, in traditional inorganic glasses such as silicates, the addition of so‐called network modifiers (typically alkali and alkaline earth oxides) is a widely used method to significantly lower the melting point [32]. This works by breaking Si‐O─Si linkages to add an excess oxygen and produce two Si─O^−^ groups, which are charge balanced by the modifier cations with weaker and less localized ionic bonding [33, 34]. Inspired by this principle, the introduction of modifiers into MOFs has been proposed as a promising approach for lowering the melting point [35, 36, 37]. For example, we have previously reported that water [36] can act as a modifier when mixed with ZIF‐62 under high temperature and pressure, lowering its melting temperature (*T*_m_) from 447°C to 295°C and its glass transition temperature (*T*_g_) from 320°C to 200°C. We have also shown that benzimidazolium and imidazolium halide salts can be used to modify ZIFs [35]. In addition to lowering the melting points of ZIF‐4 and ZIF‐62, this approach also allowed for the melting and glass formation of ZIF‐8. Recently, Kolodzeiski et al. [38] demonstrated that incorporating sodium and lithium benzimidazolate into ZIF‐62 can lower *T*_g_ from 294°C to 161°C, while Weiß et al. [39] demonstrated that co‐melting ZIF‐62 with 1,10‐phenanthroline significantly reduced its *T*_g_ from 316°C to 206°C (Co‐based system), by reducing network connectivity through formation of terminal ligands. The significance of these studies lies in the widening of the thermal processing window, i.e., the difference between the glass transition and thermal decomposition temperatures. For a given viscosity‐temperature dependence (expressed in the material's liquid fragility), this means that processing would become possible at a lower viscosity, thus opening a whole new range of potential forming techniques [40, 41, 42, 43].

To further explore and expand the concept of modifiers in ZIFs, we here investigate the use of ammonium halide salts, i.e., ammonium chloride (NH_4_Cl), ammonium bromide (NH_4_Br), and ammonium iodide (NH_4_I), as purely inorganic modifiers to reduce *T*_m_ and *T*_g_ of ZIF‐62, ZIF‐4, and ZIF‐8. We show that the incorporation of ammonium halides greatly mimic previous modification mechanisms, but as a key advantage, it allows for full evaporation of the remnants of the added modifier in the form of NH_3_. As such, our approach offers new insights into modifier‐framework interactions and opens design pathways for engineering melt‐processable MOF glasses with tunable thermal behavior and macroscopic properties.

## 2 Experimental Methods

### 2.1 Chemicals

Ammonium iodide (NH_4_I, ≥99%, p.a., ACS) was acquired from Carl ROTH. Ammonium chloride (NH_4_Cl, ≥99.5%), ammonium bromide (NH_4_Br ≥99.99%), Zn(NO_3_)_2_·6H_2_O (≥99.0%), methanol (99.9%), imidazole (99.5%), benzimidazole (98%), 2‐methylimidazole (99%), and dimethylformamide (DMF) (99.9%) were all acquired from Sigma–Aldrich. All chemicals were used as received.

### 2.2 Synthesis of ZIF‐62 Crystals

Synthesis was performed according to the procedure described in Refs. [24, 44, 45]. The samples were prepared by weighing 1.7460 g Zn(NO_3_)_2_^·^6H_2_O (5.929 mmol), 5.3282 g of imidazole (78.36 mmol), and 1.6268 g of benzimidazole (13.786 mmol) and adding them sequentially to a beaker, followed by the addition of 50 mL of DMF. The solution was stirred for 30 min, then transferred to a 100 mL PTFE‐lined autoclave and sealed. The mixture was then placed in an autoclave at 130°C for 96 h. The autoclave was allowed to cool naturally in the oven to ambient temperature overnight. The synthesized crystals were recovered and washed three times with approximately 40 mL of DMF each time. The centrifugation step was performed at approximately 7010 g (8000 rpm). The washed crystals were dried in an oven at 110°C overnight.

### 2.3 Synthesis of ZIF‐4 Crystals

The synthesis of ZIF‐4 followed the method described in Ref. [28]. 1.2 g of Zn(NO_3_)_2_·6H_2_O (4.03 mmol) and 0.9 g of imidazole (13.2 mmol) were dissolved in 75 mL of DMF and transferred to a 100 mL PTFE‐lined autoclave and sealed. The autoclave was sealed and heated in an oven at 130°C for 48 h. The synthesized crystals were recovered and washed three times with approximately 40 mL of DMF. The centrifugation step was performed at approximately 7010 g (8000 rpm). The washed crystals were dried in an oven at 110°C overnight.

### 2.4 Synthesis of ZIF‐8 Crystals

ZIF‐8 synthesis followed a procedure adapted from Ref. [46]. Initially, 0.3 g of Zn(NO_3_)_2_·6H_2_O (1.008 mmol) was dissolved in 11.3 g of methanol. Subsequently, 0.66 g of 2‐methylimidazole (8.038 mmol) and another 11.3 g of methanol were added to the zinc‐based solution. After a reaction time of 12 h, the gel formed in the solution was centrifuged at approximately 7010 g (8000 rpm) to separate the crystals. The crystals were then washed three times with methanol. The washed crystals were dried at 75°C overnight.

### 2.5 Synthesis of Modified ZIF Glasses

The preparation of the glasses was carried out in a tube furnace. Precisely weighed amounts of ZIF crystals and ammonium halide salts (total mass approximately 0.2 g) were thoroughly ground together in an agate mortar to ensure homogeneity. The specific molar ratio between ZIF and salt is described as the parameter *R*, where *R* = *n*_[modifier]_ / *n*_[ZIF]_. In this study, *R*‐values of 0.1, 0.15, 0.25, 0.5, 0.75, or 1.0 were tested to investigate the effect of salt concentration on ZIF melting and glass formation. This parameter is used throughout the remaining part of the text. The resulting powder was then pressed into pellets of 1 cm diameter under a uniaxial pressure of ∼24.5 MPa using a hydraulic press. The pellets were placed in an alumina boat and heated in a tube furnace under a continuous flow of nitrogen gas to prevent oxidation. The heating rate was set to 10 K min^−1^, followed by an isothermal dwelling period of 10 min at the target temperature to ensure complete melting. Subsequently, the samples were cooled to room temperature at the same rate. The obtained melt‐quenched materials were collected and used for subsequent structural and thermal analyses.

### 2.6 Powder X‐Ray Diffraction (PXRD)

PXRD measurements of the samples were performed on a PANalytical Empyrean X‐ray diffractometer with Cu Kα (λ = 1.5406 Å) radiation. The XRD patterns were collected in the 2*θ* range of 5°–40° with a step size of 0.013°.

### 2.7 Pair Distribution Function (PDF) Analysis

PDF measurements for ZIF‐62 crystal, ZIF‐62 glass, and ZIF‐62‐NH_4_Cl *R* = 0.5 and ZIF‐62‐NH_4_Cl *R* = 1.0 glass samples were conducted on a laboratory STOE STADI P diffractometer equipped with a silver X‐ray source. By using monochromatization (curved Ge crystal), the X‐ray beam was filtered to Ag Kα_1_ radiation (λ = 0.559407 Å). The samples were crushed and packed into special glass capillaries, 1.0 mm outer diameter, 0.01 mm wall thickness (Capillary Tube Supplies Ltd), and then flame‐sealed to prevent moisture uptake. The data was collected with the Debye‐Scherrer geometry, using two MYTHEN 2K detectors in the 2θ range of 1.350°–134.025°, with a total acquisition time of 24 h for each sample.

In addition to the above‐mentioned laboratory total scattering and PDF data, total scattering experiments for ZIF‐62 crystal, ZIF‐62 glass, and ZIF‐62‐NH_4_Cl *R* = 1.0, ZIF‐62‐NH_4_Br *R* = 1.0, and ZIF‐62‐NH_4_I *R* = 1.0 glass samples were also conducted at the Powder Diffraction and Total Scattering Beamline P02.1, PETRA III, Deutsches Elektronen‐Synchrotron DESY [47]. An X‐ray wavelength of 0.207 Å (∼60 keV) was used. The total scattering data were collected using a Varex XRD 4343CT area detector (150 × 150 µm^2^ pixel area, 2880 pixels × 2880 pixels consisting of CsI scintillators directly deposited on amorphous Si photodiodes). To extend the probing range of reciprocal space to aid the real space resolution of the resulting PDFs, the area detector was placed in ‘corner configuration’, i.e., with the X‐ray beam centered in the lower right corner of the area detector, looking from the sample position and downstream of the X‐ray beam toward the area detector. To ensure proper powder averages, capillaries were spun during acquisition. For calibration of the experimental geometry, total scattering data were acquired for LaB_6_ NIST SRM660c powder prepared in a 0.8 mm (inner diameter) special glass capillary (0.01 mm wall thickness, WJM‐Glass Müller GmbH). As mentioned above, for the laboratory total scattering measurements, special glass capillaries, 1.0 mm outer diameter, 0.01 mm wall thickness (Capillary Tube Supplies Ltd), flame‐sealed to prevent moisture uptake, were used for the samples. To allow for background‐subtraction, total scattering data were also collected for empty capillaries. The total scattering data were collected using exposure times of 300 s for LaB_6_ and 1800 s for the empty capillaries and the samples. Calibration of the experimental geometry and azimuthal integrations were done using the pyFAI library [48]. Masking was done for the beamstop, beamstop arm, and the ten outermost pixels of the area detector frame. The sample‐to‐detector distance (SDD) was calibrated to 300 mm.

The scattering data were processed by GudrunX [49] and normalized to ${<F>}^{2}$, using the Breit‐Dirac factor of 2.5, and with the Krogh–Moe and Norman normalization enabled, using the obtained elemental composition and estimated densities, resulting in a *Q*_max_ = 19.5 Å^−1^ for the laboratory silver X‐ray source and *Q*_max_ = 20.5 Å^−1^ for the P02.1 synchrotron measurements. The compositions of the composite samples were estimated based on the ZIF‐62 to halide ratio, assuming that halide incorporation leads to protonation of the linker (Im or bIm), thereby ensuring a charge‐stabilized system. The remaining NH_3_ was assumed to have completely evaporated. To further correct the background, the contribution for unphysical peaks located at low distance *r* (<1.0 Å) was suppressed; moreover, the Top‐hat function width was set to 3 Å^−1^ as an additional filter for the Fourier Transform (FT) to real space. To minimize termination ripples from the finite *Q*_max_, a Lorch‐like modification function was applied in the FT to real space (width of broadening 0.1 Å and broadening power 0.1) [49]. For the ZIF‐62‐NH_4_I sample with *R* = 1.0, a pronounced background contribution resembling fluorescence was observed. Although iodine fluorescence is not expected to significantly contribute at an incident energy of ∼60 keV, an absorption/fluorescence correction was nevertheless applied to substantially reduce the background contribution.

### 2.8 Thermogravimetric Analysis Coupled With Differential Scanning Calorimetry (TGA‐DSC)

TGA‐DSC measurements were performed using a Netzsch STA F449 F3 instrument equipped with liquid N_2_ cooling. Measurements were performed in a Netzsch sealed aluminum crucible in a He atmosphere, except for measurements conducted to obtain isobaric heat capacity, which were done in PtRh crucibles. Heating and cooling rates of 10 K min^−1^ were used. The thermal history of the as‐prepared glass was ‘reset’ by heating to 200°C (i.e., >>*T*_g_) before cooling at a rate of 10 K min^−1^ and finally acquiring a heating scan at 10 K min^−1^, which is then reported in the following. Measurements of absolute heat capacities were obtained using a sapphire as a standard material.

### 2.9 Fast Scanning Differential Calorimetry

Fast scanning differential calorimetry (FDSC) measurements were performed on selected samples using a Mettler Toledo Flash DSC 2+. Small grains of selected premelted glass samples were crushed using a scalpel blade before being loaded onto a pre‐conditioned and pre‐corrected UFS1 sensor using a hair. The samples were then prepared at approximately 250°C, to establish a good thermal contact between the sensor and the sample and remove any traces of water. All sample scans were performed in a nitrogen atmosphere (flow of 20 mL min^−1^). All heat flow traces were corrected using a symmetric approach as described in, e.g., Ref. [50].

To extract the fictive temperatures of the system, we employed the method of Moynihan et al. [51]. To this end, we performed experiments at varying cooling rates (10, 50, 100, 200, 500, 1000, and 2000 K s^−1^) followed by an upscan with a constant heating rate (1000 K s^−1^). From these measurements, we extracted the *T*_f_ and subsequently the activation energy for viscous flow as [52]:

$$\mathrm{In}\left(Q\right)=-\frac{E_{g}}{RT_{f}}+c$$

from which the fragility could be obtained as:

$$m=\frac{E_{g}}{RT_{g}\ln\left(10\right)}$$

Here, we used the values of *T*_g_ obtained from the standard DSC measurements, i.e., 80 °C for ZIF‐62‐NH_4_Cl *R* = 1 and ZIF‐62‐NH_4_I *R* = 1 samples, as well as 133°C for the ZIF‐62‐NH_4_Cl *R* = 0.5 sample.

### 2.10 Scanning Electron Microscopy (SEM)

The surface morphology and elemental distribution of the samples were investigated using a field‐emission scanning electron microscope (FE‐SEM, ZEISS Sigma 300, Germany). Small amounts of bulk samples were directly mounted onto aluminum stubs using conductive carbon tape. Prior to imaging, a thin gold coating was applied using a HEZAO LAB GMC‐1500 sputter coater at a current of 10 mA for 45 s. The sputtering time was adjusted depending on the sample type and measurement requirements. SEM imaging was performed under high vacuum using an SE2 secondary electron detector. For morphology observations, an accelerating voltage of 3 kV was applied. For energy‐dispersive X‐ray spectroscopy (EDS) mapping, the accelerating voltage was set to 15 kV to ensure adequate excitation of characteristic X‐rays.

### 2.11 Fourier Transform Infrared (FT‐IR) Spectroscopy

FT‐IR spectra were recorded using an attenuated total reflection setup on a Bruker Tensor II spectrometer. Diamond was used as the attenuation crystal. All samples were measured under ambient conditions in the 400–4000 cm^−1^ frequency range.

### 2.12 Far‐Infrared (FIR) Spectroscopy

Far‐infrared spectra were collected using a Thermo Fisher Scientific Nicolet iS50 FTIR spectrometer equipped with a far‐IR beam splitter and detector module. Powdered samples were finely ground and homogeneously mixed with paraffin oil, then spread onto a polyethylene (PE) film for measurement. Spectra were acquired in the far‐infrared region from 450 to 100 cm^−1^, with appropriate resolution and scan averaging to ensure signal quality.

### 2.13 Liquid‐State ^1^H Nuclear Magnetic Resonance (^1^H NMR)

Solution ^1^H NMR spectra of digested samples (in a mixture of DCl (35%)/D_2_O (0.1 ml) and dimethyl sulfoxide‐d_6_ (DMSO‐d_6_; 0.5 ml)) were performed [53]. In detail, spectra of desolvated crystalline samples and glasses (about 10 mg each) were recorded on a Bruker Avance III 600 MHz spectrometer at 308 K. Chemical shifts were referenced to the residual solvent signals of non‐deuterated DMSO. The spectra were processed with the MestreNova Suite.

### 2.14 X‐Ray Photoelectron Spectroscopy (XPS)

XPS measurements were carried out using a Thermo Scientific K‐Alpha system equipped with a monochromatic Al Kα_1_ radiation source (*hν* = 1486.6 eV). The system operated at an accelerating voltage of 12 kV and a filament current of 6 mA. The pressure in the analysis chamber was maintained below 2.0·10^−7^ mbar throughout the measurements. Samples (including powders and bulk fragments) were mounted onto sample holders using double‐sided conductive tape. Powdered samples were gently pressed to form pellets before mounting, while film and bulk specimens were cut to appropriate sizes prior to analysis. To minimize sample charging, a dual‐beam charge compensation system was employed. The spot size for analysis was set to 400 µm. For survey scans, a pass energy of 150 eV and a step size of 1.0 eV were used, whereas high‐resolution scans were acquired with a pass energy of 50 eV and a step size of 0.1 eV. The exact acquisition parameters were optimized depending on the sample.

### 2.15 X‐Ray Imaging

X‐ray imaging experiments were performed to further characterize the co‐melting process. Prior to this, ZIF‐62 crystals were physically stacked with NH_4_Cl crystals. The synchrotron radiation experiment was carried out on the BL13W1 beamline at Shanghai Synchrotron Radiation Facility, China (SSRF). The main surface of the sample was set perpendicular to the incident monochromatic X‐ray beam with an energy of 30 keV. The time‐sequenced images were captured by a charge coupled device (CCD) with a resolution ratio of 3.25 µm per pixel [54]. The sample was heated to 360°C at a heating rate of ∼20.5°C min^−1^ with an accuracy of ±1°C. After reaching 360°C, the temperature was held constant. Each image was captured over 0.2 s with a 1 s interval between successive frames.

## 3 Results and Discussion

### 3.1 Glass Formation

**FIGURE 1:**
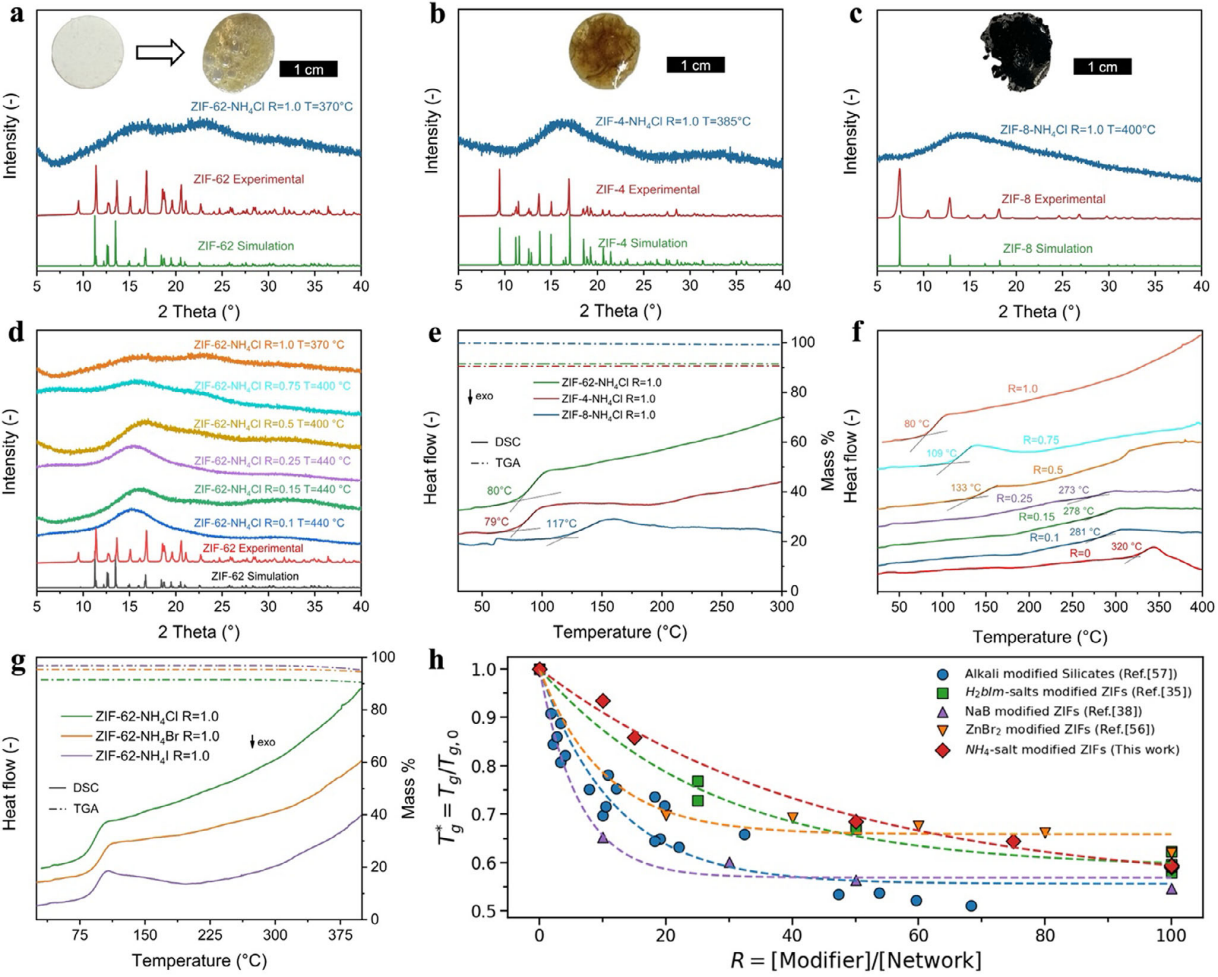
(a‐c) X‐ray diffraction patterns of studied ZIF materials, including simulated (CCDC deposition numbers: ZIF‐4: 602538, ZIF‐8: 1429243, and ZIF‐62: 671070) [55] and experimental crystalline forms and experimentally formed glasses upon co‐melting with ammonium chloride (*R*  =  1.0) at different temperatures for (a) ZIF‐62, (b) ZIF‐4, and (c) ZIF‐8. The insets show photographs of pressed pellets of ZIF crystal mixed with ammonium halide salt before and after heating (scale bar represents 1 cm). (d) X‐ray diffraction patterns of melt‐quenched products of ZIF‐62‐NH_4_Cl with varying *R* from 0.1 to 1.0. (e‐g) DSC and TGA heating traces at 10 K min^−1^ of (e) ZIF‐62‐NH_4_Cl, ZIF‐4‐NH_4_Cl, and ZIF‐8‐NH_4_Cl (*R* = 1.0), (f) ZIF‐62‐NH_4_Cl (*R* from 0 to 1.0), and (g) ZIF‐62‐NH_4_X (X=Cl, Br, and I; all *R* = 1.0) hybrid glasses. (h) Dependence of the reduced glass transition temperature ($T_{g}^{*}=T_{g}\;T_{g,0}^{-1}$, where *T*_g_ is the glass transition of a modified glass and *T*_g,0_ is the glass transition temperature of the pure glass former, here ZIF‐4 and ZIF‐62) on the modifier ratio. Results are shown for the present ammonium halide‐modified ZIF‐4 and ZIF‐62 glasses, sodium benzimidazolate (NaB) modified ZIFs [38], benzimidazole halide‐modified ZIFs [35], ZnBr_2_ modified ZIFs [56], and alkali‐modified silicate glasses [57]. The dashed lines represent exponential fits to the data and serve as guides to the eye.

In this study, we use ammonium halide salts (NH_4_Cl, NH_4_Br, and NH_4_I) as chemical modifiers to facilitate the melting and glass formation of ZIF‐4, ZIF‐62, and ZIF‐8. First, X‐ray diffraction analysis confirms the formation of the correct starting ZIF crystals, as shown in Figure 1a–c. The salts are introduced at various molar ratios (*R*) into these ZIF crystals, where *R* = *n*_[modifier]_ / *n*_[ZIF]_, and the mixtures are subjected to thermal treatment in a tube furnace followed by melt‐quenching (see Methods section) to obtain glassy products, as shown in the insets of Figure 1a–c and Figure S1. PXRD confirms that all melt‐quenched products transformed into fully non‐crystalline states (Figure 1a–c; Figure S2). For the melt‐quenched ZIF‐62‐NH_4_Cl *R* = 1.0 glass (inset of Figure 1a), some bubble formation is observed, which might be attributed to the release of ammonia originating from the ammonium salts. Interestingly, for the same *R* value, samples modified with NH_4_Br and NH_4_I exhibit much fewer bubbles compared to those modified with NH_4_Cl (Figure S1a), suggesting that the nature of the halide anion plays a critical role in influencing the melting behavior, possibly due to variation in melt viscosity. The melting behavior of the ZIF‐62‐NH_4_Cl system is also found to depend on the modifier concentration (Figure 1d and Figure S1b,c). Notably, at low salt content (*R* ≤ 0.25), a relatively high temperature of 440°C is required to achieve complete melting (loss of crystallinity). As the NH_4_Cl concentration increases, the melting temperature gradually decreases, dropping to 400°C at *R* = 0.5‐0.75 and further to 370°C at *R* = 1.0.

The obtained MOF glasses are further analyzed using standard differential scanning calorimetry (DSC) measurements. The glass transition temperature (*T*_g_) is identified as the onset temperature of the glass transition [58], as shown in Figure 1e–g. We find that NH_4_Cl‐modified systems (ZIF‐4, ZIF‐62, and ZIF‐8, all *R =* 1.0) exhibit clear glass transition regions in the range of 70–120°C (Figure 1e). Upon changing the modifier content (*R*), *T*_g_ of ZIF‐62‐based glasses decreases with increasing *R* (see Figure 1f and Figure S3a). Specifically, *T*_g_ decreases from 320°C for the pristine ZIF‐62 (*R* = 0) to 281°C at *R* = 0.1, 273°C at *R* = 0.25, and further to 130°C at *R* = 0.5, eventually reaching 80°C at *R* = 1.0. This suggests that the incorporation of NH_4_Cl disrupts the original glass network, leading to a more flexible amorphous structure. Similar reductions in *T*_g_ have been observed in ZIF‐62 glasses modified with NH_4_Br and NH_4_I (Figure 1g), suggesting that halide anions have a significant effect on the overall *T*_g_ suppression behavior. A comparable *T*_g_‐depressing trend is also found for ZIF‐4 modified with ammonium halide salts (Figure S3b), confirming the generality of this modifier‐induced softening effect across different ZIF systems.

To further understand the salt‐ZIF interaction, we homogenously mix ZIF‐62 crystals with NH_4_Cl at *R* = 1.0 and subject them to three consecutive heating cycles. As shown in Figure S4, a broad endothermic feature is observed during the first heating scan. This signal is accompanied by a noticeable mass loss, indicating potential decomposition or volatilization of labile species. We interpret this as the release of adsorbed water (starting at ∼100 °C) as well as the reaction between the modifier and the ZIF, in which thermal activation promotes partial disruption of Zn─N coordination bonds, likely facilitated by halide anion coordination and the release of volatile NH_3_ from the decomposition of NH_4_^+^. This interaction initiates depolymerization of the framework and leads to the formation of a hybrid liquid and a glass upon subsequent cooling. In contrast, the second and third heating cycles display a sharp and reproducible transition ∼75°C, without any associated mass loss, characteristic of a glass transition. Notably, this *T*_g_ value is close to that measured for the melt‐quenched glass from tube furnace treatment (∼80°C), further validating the formation of a stable glass through this route. Compared to the sodium benzimidazolate‐modified ZIF‐62 glasses by Henke et al. (*T*_g_ of 162 to 192°C) [38], the *T*_g_ values observed herein are significantly lower, but slightly higher than those reported for ZIFs modified with organic benzimidazole salts (*T*_g_ of 30°C–35°C) [35], and is comparable to that of melt‐quenched ZnX_2_(bIm)_2_ glasses (X = Cl, Br, and I) with *T*_g_ of 82°C–91°C [59].

Overall, our observations thus align with the common modification principle in silicate glasses, with decreasing *T*_g_ for increasing modifier content (increasing *R* value). We summarize the present data with our earlier work on benzimidazole‐based halide salts [35], recent work on modification of ZIFs from literature [38], ZnBr_2_ modified ZIFs [56], and a range of silicate glasses [57] in Figure 1h, finding that all modified glass systems exhibit a consistent trend of decreasing *T*_g_ values with increasing modifier content. In this regard, we infer that ammonium halides enable a “clean” ionic modification of ZIFs, distinct from alkali metal benzimidazolates [38] and ionic liquids [8], which show low stability and/or deposition of species that are prone to decompose. In our approach, upon heating, the halide salts melt and infiltrate the ZIF crystal, the halide anions (X^−^) substitute Zn─N bonds to form stable Zn─X bonds, while NH_4_^+^ decomposes into volatile NH_3_ that fully leaves the system. This mechanism disrupts the Zn─N network, giving rise to a lower *T*_g_ (from 320°C of ZIF‐62 glass to 75°C–80°C of modified glasses), while preserving thermal stability (*T*_d_), and thereby maximizing the processing window (*T*_d_–*T*_g_) and enabling high‐purity, melt‐processable ZIF glasses. In addition to the regular DSC measurements, we have also performed fast scanning differential calorimetry (FDSC), enabling much faster scanning rates than standard DSC (up to 2.4·10^6^ K min^−1^). The results are shown in Figures S5 and S6. Based on these, we confirm the good glass‐forming abilities and also extract liquid fragilities values of *m* = 40 for ZIF‐62‐NH_4_Cl *R* = 0.5, while it increases to *m* = 46 for *R* = 1. Exchange of the halide ion only marginally changes the fragility to *m* = 42 for ZIF‐62‐NH_4_I *R* = 1. In comparison, the pure ZIF‐62 glass has a very low fragility of ∼23 [60]. This increase of *m* with increasing modifier content has previously been observed for modified silicate glasses as well as in another recent study on a modified ZIF system [38, 56, 62].

### 3.2 Sample Morphology and Homogeneity

The reduced melting points of the ammonium halide‐modified ZIFs allow for easier processing. As shown in the insets of Figure 1a–c and Figure S1, several of the obtained glasses display dimensions exceeding 1 cm. This is difficult to obtain for pure ZIF glasses, which often suffer from a large degree of defects [19]. For example, melt‐quenched ZIF‐4 glass yields small pieces (only a few millimeters) due to foaming, while ZIF‐8 decomposes before melting. Although previous attempts using ionic liquids can reduce the melting point of ZIF‐8 to below its decomposition temperature (∼600°C), the resulting glass fragments are <1 mm in size [8]. Here, the incorporation of NH_4_Cl, NH_4_Br, or NH_4_I enables the formation of bulk ZIF‐8‐derived glasses that are fully non‐crystalline (yet black‐colored, likely due to minor decomposition) regardless of halide type. This is reminiscent of the sample size and quality obtainable using benzimidazolium‐based halide salts [35]. However, unlike the low‐temperature melting (typically below 300°C) enabled by the organic salts, the present method using inorganic salts (NH_4_Cl/Br/I) requires higher processing temperatures (370°C–400°C). Nevertheless, the successful formation of macroscopic glass indicates a significant stabilization effect imparted by ammonium halides during the melt‐quenching process, and the resulting higher *T*_g_‐values of ammonium relative to benzimidazolium‐based halide salts provide improved thermal stability for applications.

**FIGURE 2:**
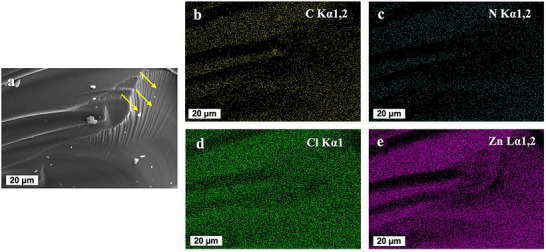
SEM‐EDX elemental mapping of the ZIF‐62‐NH_4_Cl glass with *R* = 1.0, showing the imaged (a) sample surface (yellow arrows highlight the smooth viscous flow and fracture lines) and the corresponding spatial distribution of (b) C, (c) N, (d) Cl, and (e) Zn elements. Scale bars are displayed in the bottom left corner of each image.

The surface morphology and compositional homogeneity of a representative modified glass (ZIF‐62‐NH_4_Cl, *R* = 1.0) is further characterized by scanning electron microscopy (Figure 2a). We observe typical morphological features from the liquid state, including viscous flow and fracture lines typical of glasses, since the surface is smooth and uniform. Elemental mapping results obtained using energy‐dispersive X‐ray spectroscopy are shown in Figure 2b–e (EDX map sum spectrum is shown in Figure S7). We find a homogeneous distribution of C, N, Cl, and Zn elements at the microscopic level. Similar compositional uniformity is observed for a lower modifier content of *R* = 0.5 (Figure S8), and in samples modified with NH_4_Br (Figure S9) and NH_4_I (Figure S10) as well as modified glasses derived from ZIF‐4 (Figure S11) and ZIF‐8 (Figure S12).

### 3.3 Structure Characterization

**FIGURE 3:**
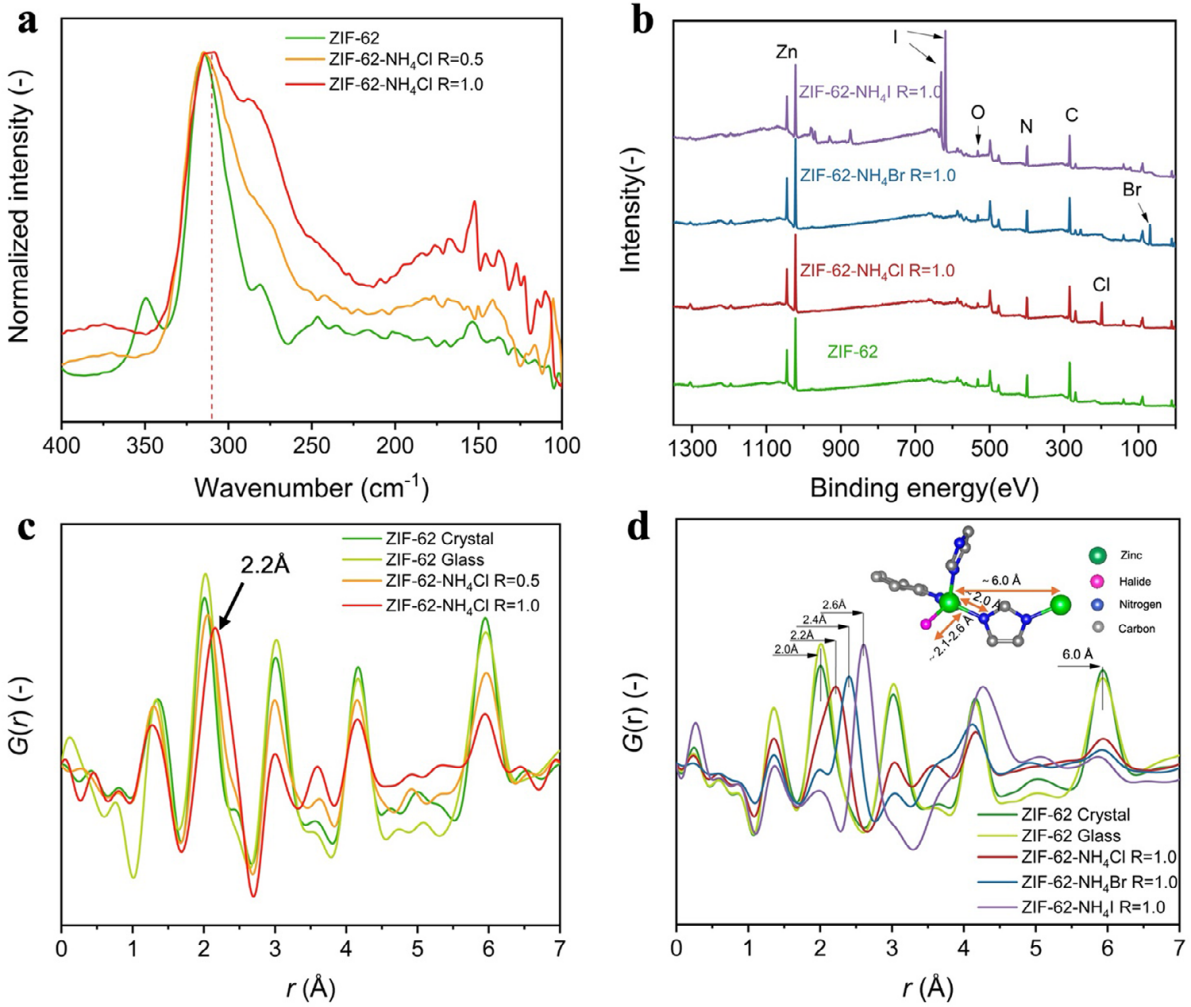
(a) Far‐infrared absorbance spectra of ZIF‐62‐NH_4_Cl glasses with different *R* values (0.5 and 1.0). (b) X‐ray photoelectron spectroscopy data of ZIF‐62 crystal and ZIF‐62‐NH_4_X glass at *R* = 1.0 and X=[Cl, Br, and I]. (c,d) All reduced pair distribution function *G*(*r*) are obtained with a Lorch‐like modification function and a *Q*_max_ = 19.5 Å^−1^ for (c) ZIF‐62 crystal and glass as well as ZIF‐62‐NH_4_Cl glasses (*R* of 0.5 and 1.0) (data from a laboratory diffractometer equipped with a silver X‐ray source) and (d) ZIF‐62 crystal and glass as well as ZIF‐62‐NH_4_Cl, ZIF‐62‐NH_4_Br, ZIF‐62‐NH_4_I glasses (data from Total Scattering Beamline P02.1, PETRA III, Deutsches Elektronen‐Synchrotron DESY). Note that data is only shown in the range of 0–7.0 Å.

After confirming the glassy nature of the modified ZIF glass materials, we characterize their atomic‐scale structure. First, we examine the vibrational features of the modified glasses using FT‐IR and FIR spectroscopy. The FT‐IR spectra (Figure S13) show that the characteristic vibrational modes of the zeolitic imidazolate framework are largely preserved upon modification with different ammonium halide salts. For the ZIF‐62 system, increasing the *R* value of NH_4_Cl from 0.1 to 1.0 (Figure S13a) does not result in significant changes in the characteristic vibrational bands. Similar results are observed for ZIF‐62 modified by NH_4_Br and NH_4_I (Figure S13b), as well as the derivative glasses of ZIF‐4 and ZIF‐8 (Figure S13c,d). This suggests that the incorporation of ammonium halide salts does not compromise the structural integrity of the organic linkers in the ZIF structure (which comprise the main bands of the FT‐IR spectra). To further investigate the local bonding environment and especially the metal‐ligand interactions, FIR spectra have also been collected (Figure 3a and Figure S14). In the NH_4_Cl‐modified ZIF‐62 glasses, this peak becomes more intense with increasing *R* (0, 0.5, and 1.0) (Figure 3a), and additional broad features emerge at 310 cm^−1^. We ascribe these changes to the formation of new coordination environments, likely involving Zn‐halide interactions. These spectra probe the low‐frequency region (100–400 cm^−1^), which corresponds to frequencies of Zn─N and Zn─X (X = Cl, Br, and I) vibrational modes. We find a prominent peak around 310–315 cm^−1^, which we assign to Zn─N stretching, likely with an overlap of vibrations from Zn─Cl [56, 63, 64]. Additional peaks are observed at ∼230 cm^−1^, corresponding to Zn─Br [65], while the peak in the range of 150–200 cm^−1^ is tentatively attributed to Zn─I vibrations.

Next, X‐ray photoelectron spectroscopy is used to investigate the surface chemical composition and bonding environment of the modified ZIF‐derived glasses. Survey spectra of ZIF‐4, ZIF‐8, and ZIF‐62 samples before and after NH_4_X (X = Cl, Br, and I) modification at *R* = 1.0 clearly show the presence of halogen element peaks in the modified samples (Figure 3b and Figure S15). Additionally, a noticeable increase in signal intensity is observed for C 1s and N 1s peaks after modification, indicative of changes in the local electronic environment or partial framework disruption. High‐resolution spectra further reveal subtle shifts in the binding energies of C 1s and N 1s peaks (Figures S16 and S17). Based on peak fitting, we find that after NH_4_X (X = Cl, Br, and I) modification, a new N 1s peak appears at ∼399.3 eV, which we attribute to protonated N─H^+^ species (in benzimidazolium or imidazolium) or trace NH_4_^+^, indicating proton transfer and an altered coordination environment [66]. In contrast, the Zn 2p doublet shows no significant change upon modification, suggesting that the oxidation state and core Zn coordination environment remain intact, but overall, these shifts suggest coordination interactions between halide ions and the Zn centers or surrounding ligands [67]. Furthermore, the detection of O 1s peaks in all modified samples suggests either minor oxidation during processing or possibly moisture uptake on the glass surface.

To further probe the structural integrity of the organic linkers after NH_4_Cl modification (*R* = 1.0), liquid‐state ^1^H NMR spectroscopy measurements have been conducted. As shown in Figure S18, the characteristic proton resonances corresponding to imidazolate, benzimidazolate, and methyl‐imidazolate linkers (depending on the type of ZIF) originating from digested samples (see Methods) remain well‐resolved and unchanged upon modification, suggesting there is no significant ligand decomposition or exchange. Notably, a new signal appearing between 7.5 and 8.0 ppm is observed (marked with a black arrow), which might be attributed to free ammonium ions (NH_4_^+^) present in solution. Overall, we infer that the melting of ZIFs with ammonium halide salts does not compromise the integrity of the organic linkers at the molecular level.

We then characterize the local Zn environments upon modifier incorporation through X‐ray total scattering and atomic pair distribution function (PDF) analysis of the melt‐quenched ZIF‐62‐NH_4_Cl glasses with *R* values of 0, 0.5, and 1.0. PDF results are shown in Figure 3c, while the reciprocal space structure factor *S*(*Q*) data are shown in Figure S19a. The PDF analysis provides direct knowledge on the interatomic distances present in the material, even for fully amorphous materials [68]. That is, the local structural order at short distances (*r*< 8 Å) is observed as fluctuating deviations from the average number density (*ρ*_0_), whereas at longer distances (*r* >8 Å), the amorphous samples follow *ρ*_0_ due to the lack of periodicity. From the PDF analysis, we observe a shift of the Zn─N peak located at approximately 2.0 Å [69] to around 2.2 Å as the *R* value increases. Given the difference in size of N and Cl atoms, this points to the formation of Zn─Cl bonds [70, 71] and that chloride ions are incorporated into the tetrahedral Zn coordination environments during the melt‐quenching process. We also probe the structural influence of different halide modifiers (Br^−^ and I^−^), as shown in Figure 3d (*S*(*Q*) data are shown in Figure S19b). We find distinct local coordination distances associated with different halogen species. Specifically, we find evidence of Zn─Br and Zn─I bonds at distances of approximately 2.4 and 2.6 Å [72, 73] for the glasses modified using NH_4_Br and NH_4_I, respectively. The Zn─X (X = Cl, Br, I) bond distances show a monotonic increase with the halide ionic radius (Cl^−^:181 pm, Br^−^:195 pm, I^−^:216 pm). This structural evolution originates from the gradual expansion of the Zn‐centered tetrahedral coordination sphere with the increase of the halide ion size. That is, the larger halide anions occupy the coordination sites of the original Zn─N network, leading to a gradual increase in the average bond length of the Zn coordination shell. Interestingly, this observation is consistent with our previous results [35] on benzimidazolium halide‐modified ZIFs. These results clearly demonstrate that the incorporation of halide anions leads to distinct Zn─X (X = Cl, Br, and I) bond distances, reflecting the progressive modification of the Zn coordination environment depending on the halogen species.

In addition, the glass transition temperatures of the ZIF‐62 glasses modified with ammonium salts (at *R* = 1.0) are 80°C, 78°C, and 75°C, respectively (see Figure 1g), showing a slight decrease in *T*_g_ with increasing halide ionic radius and Zn─X bond length. This trend suggests that larger halides (e.g., I^−^) induce a looser local Zn coordination environment and greater disruption of the tetrahedral network, reducing network rigidity and thus lowering *T*_g_. Conversely, a smaller Cl^−^ causes a milder disruption and a more compact structure, resulting in a slightly higher *T*_g_. This is similar to what has been observed in oxide glasses, where larger network‐modifier cations have lower “field strength” and thus less network‐forming character [74], as well as the trend observed when exchanging oxygen for sulfur [75]. Here, we observe similar structural trends for modified ZIF‐62, ZIF‐4, and ZIF‐8 glasses (Figure S20a–d). Although the incorporation of halide salts generates non‑bridging imidazolate linkers and terminal Zn─X bonds, previous CO_2_ adsorption measurements on similarly modified ZIF glasses reveal a clear decrease in CO_2_ uptake capacity with increasing modifier content [35]. To further study this, we believe that advanced measurements using, e.g., positron annihilation spectroscopy [76] or NMR measurements of adsorbed ^13^CO_2_ [77] are needed to access the concentration of defective sites.

### 3.4 Modifer Facilitated Melting

**FIGURE 4:**
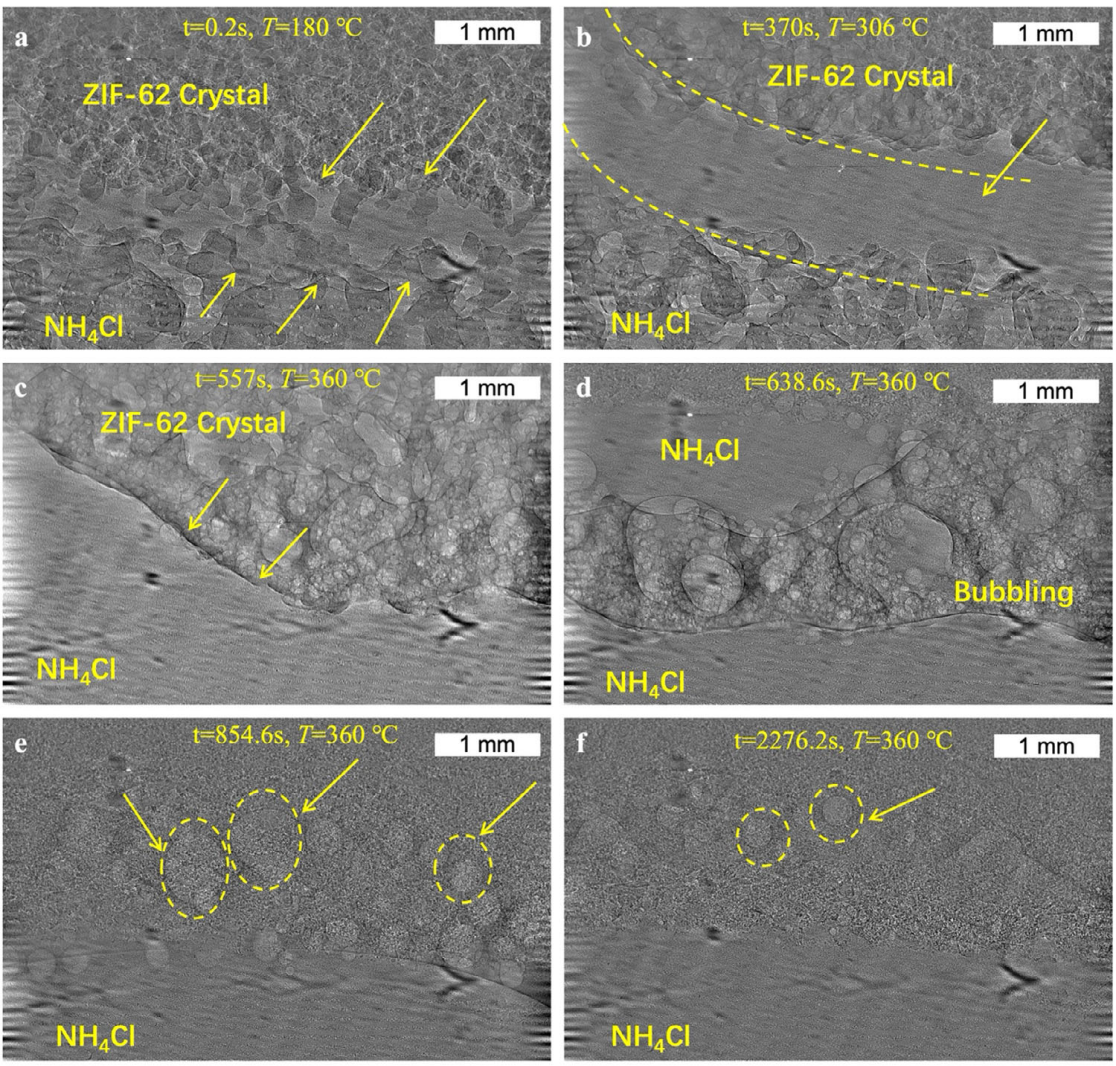
X‐ray phase‐contrast imaging showing the sequential co‐melting process of ZIF‐62 crystals and NH_4_Cl. (a) At a temperature of 180°C, a distinct boundary is observed between the ZIF‐62 crystals and NH_4_Cl particles (arrows point to the interface between these crystals). (b) Upon heating to 306°C, NH_4_Cl begins to melt, resulting in a gradual blurring and broadening of the interface and initial wetting of the ZIF‐62 crystal surface. (c) With further temperature increase to 360°C, NH_4_Cl becomes fully molten and starts to partially infiltrate the ZIF‐62 crystal region (arrows point to the boundary of the NH_4_Cl liquid surface). (d) Extended heating at 360°C leads to encapsulation of the ZIF‐62 crystals by molten NH_4_Cl, accompanied by bubble formation. (e) Continued heating to 360°C leads to the onset of ZIF‐62 melting, characterized by the softening of the crystal edges and increased bubble formation (arrows and dashed circles indicate bubbles). (f) Upon complete melting of ZIF‐62‐NH_4_Cl 360°C, most bubbles have been released, leaving only a few trapped within the melt, indicating the formation of a homogeneous liquid phase. Scale bars are displayed in the upper right corner of each image.

To understand the salt‐promoted melting behavior of the ZIFs, we have performed in situ X‐ray imaging experiments to visualize the macroscopic (mm‐scale) mixing of the ZIF‐62 crystal in the presence of NH_4_Cl (Figure 4) during the melting process. Initially, we observe a distinct interface between the two crystalline phases, with ZIF‐62 crystals and NH_4_Cl particles being clearly separated (Figure 4a). Upon heating, we then observe how NH_4_Cl begins to melt (∼300°C), resulting in a gradual broadening of the interfacial boundary (Figure 4b). As the heating continues to *T* = 360°C, NH_4_Cl becomes fully molten and partially merges with the interstitial regions of the ZIF‐62 crystals (Figure 4c). Eventually, the NH_4_Cl liquid phase fully encapsulates the ZIF‐62 crystals, accompanied by the generation of gas bubbles (likely NH_3_) at the interface (Figure 4d), suggesting potential volatilization or interfacial reactions. Continued heating at *T* = 360°C leads to the melting of the ZIF‐62 crystals themselves (Figure 4e), followed by the disappearance (release) of most gas bubbles and formation of a homogeneous melt (Figure 4f). This sequence of events visually confirms that molten NH_4_Cl plays a facilitative role in promoting the melting of ZIF‐62 crystals by disrupting the coordination network and lowering the energy barrier for the solid‐to‐liquid transition.

To characterize the kinetics of the co‐melting process, we further analyze the in situ X‐ray imaging data as the temperature increases from 286°C to 343°C (during a time period of 168 s). That is, we measure the thickness of the interfacial region between ZIF‐62 and NH_4_Cl, as shown in Video S1. The growth of the interfacial reaction layer proceeds unilaterally into the ZIF‐62 crystal upon heating, and its thickness increases linearly with time (Figure S21). As such, we extract the growth rate of the interfacial region using a sliding‐window linear fitting approach. Within the investigated temperature range, we find the rate to be approximately constant with a value of ∼6.1 µm s^−1^, suggesting that the co‐melting is not controlled by a single thermally activated step.

### 3.5 Structural Modification Mechanism

**FIGURE 5:**
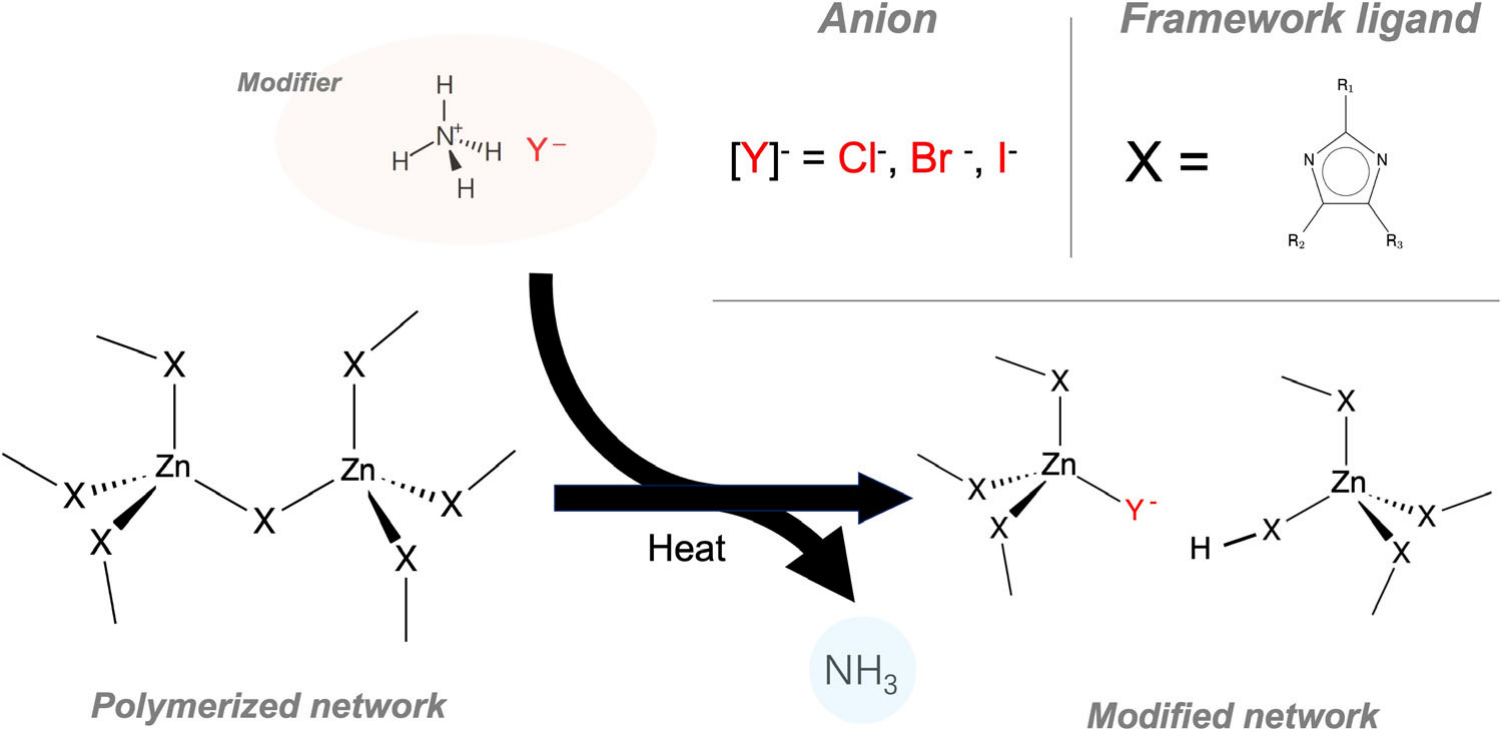
Proposed reaction scheme mechanism of ammonium halide salt modification of ZIFs.

Based on our combined experimental results and recent results for benzimidazolium and imidazolium halide salt modification of ZIFs [35], we propose a reaction scheme mechanism for the modification of ZIFs by ammonium halide salts (Figure 5). Upon heating, the ammonium halide crystal melts to form a liquid phase that physically wets and spreads over the surface of the ZIF crystals, akin to what batched carbonates do in classical silicate glass production [78]. This wetting and subsequent infiltration (mixing) process is likely driven by capillary force and favorable interfacial interactions. In the molten state, the halide salts promote the weakening or breaking of the Zn─N bonds. Simultaneously, thermal decomposition of NH_4_^+^ generates volatile species such as NH_3_, which escapes from the system, as indicated by the formation of gas bubbles observed during in situ imaging (Figure 4d). The remaining proton (H^+^) may associate with basic sites in the system (e.g., framework N atoms) or participate in further reactions. This disruption of the coordination network facilitates the depolymerization of the ZIF, and upon continued heating, a fraction of the organic linkers may escape the system, accompanied by partial repolymerization of the framework structure. In the final stage of melting, the crystalline domains collapse and bubble formation is reduced (Figure 4f), consistent with the removal of unstable gaseous components (NH_3_) and the formation of a new coordination environment (Zn─Cl, Zn─Br, or Zn─I). The preserved integrity of the organic linkers, as confirmed by FT‐IR (Figure S13) and liquid‐state ^1^H NMR spectroscopy (Figure S18), indicates that the decomposition is limited to ionic and coordination changes rather than ligand degradation.

## 4 Conclusions

In this study, we have systematically investigated the use of ammonium halide salts (NH_4_X, X = Cl, Br, and I) to chemically modify zeolitic imidazolate frameworks (ZIFs) and thereby lower their melting and glass transition temperatures. Upon heating, the halide salt melts and interacts with the ZIF, leading to the weakening of Zn─N bonds and the subsequent depolymerization of the coordination network. This is followed by halide ion incorporation in the Zn‐tetrahedra, resulting in the formation of mixed‐linker MOF glasses. The proposed modifier method is found to be broadly applicable across various ZIF systems, including ZIF‐4 and ZIF‐62 as well as the otherwise unmeltable ZIF‐8. These findings not only extend our fundamental understanding of how halide salts can act as chemical modifiers in ZIFs but also provide a promising pathway for designing melt‐processable MOF glasses with tunable properties for potential applications in, e.g., optics and separation technologies.

## Conflicts of Interest

None of the authors has a conflicts of interest to disclose.

## Supporting information




**Supporting File 1**: smll73675‐sup‐0001‐SuppMat.pdf.

**Supporting File 2**: smll73675‐sup‐0002‐VideoS1.docx.

## Data Availability

The data that support the findings of this study are available from the corresponding author upon reasonable request.
