# Supplementary material for: Tailoring the Melting and Glass Transition Behavior of Zeolitic Imidazolate Frameworks via Ammonium Halide Salts
Source: Small. 2026 May 8;22(36):e73675. doi: 10.1002/smll.73675 (PMC13306924; doi:10.1002/smll.73675)
Supplement: Supplementary file 1 — Supporting File 1: smll73675‐sup‐0001‐SuppMat.pdf. [file SMLL-22-e73675-s001.pdf]

SUPPLEMENTARY INFORMATION

for

Tailoring the Melting and Glass Transition Behavior of Zeolitic Imidazolate Frameworks via Ammonium Halide Salts

Fengming Cao[1], Søren S. Sørensen[1,], Anders K. R. Christensen[1], Xuan Ge[2], Martin A. Karlsen[3], Giulio Monaco[4], Lothar Wondraczek[5], Morten M. Smedskjaer[1,*]*

[1]Department of Chemistry and Bioscience, Aalborg University, Aalborg DK-9220, Denmark

[2]Shanghai Key Laboratory of Materials Laser Processing and Modification, School of Materials Science and Engineering, Shanghai Jiao Tong University, 200240 Shanghai, PR China

[3]Deutsches Elektronen-Synchrotron DESY, Notkestr. 85, 22607 Hamburg, Germany

[4]Department of Physics and Astronomy `Galileo Galilei', University of Padova, 35131 Padova, Italy

[5]Otto Schott Institute of Materials Research, Friedrich Schiller University Jena, 07743 Jena, Germany

[*]Corresponding authors. E-mail: soe@bio.aau.dk (S.S.S.), mos@bio.aau.dk (M.M.S.)

Supplementary Figure 1

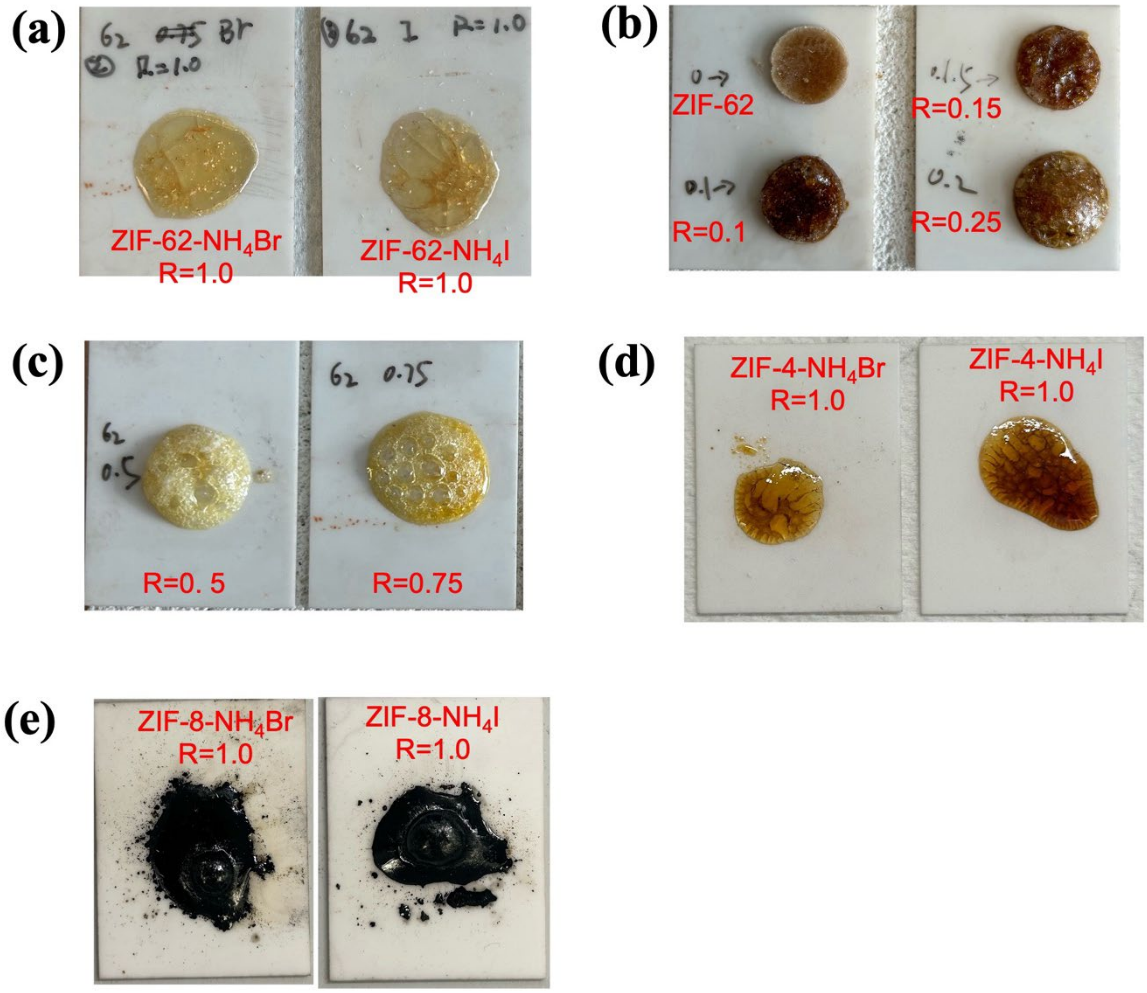

Supplementary Figure 1. Photographs of modified ZIF-derived glasses, with the width of the alumina support sheet being 2.9 cm for scale. (a) Glasses obtained from ZIF-62 combined with NH_4Br and NH_4I at R = 1.0, T=370 °C. (b-c) Glasses obtained from ZIF-62 combined with NH_4Cl at (b) R = 0 (pristine ZIF-62), 0.1, 0.15, and 0.25 were quenched after melting at 440 °C, and (c) R = 0.50 and 0.75, T=400 °C. (d) Glass obtained from ZIF-4 combined with NH_4Br/I at R = 1.0 T=385 °C. (e) Glass obtained from ZIF-8 combined with NH_4Br/I at R = 1.0 T=400 °C.

Supplementary Figure 2

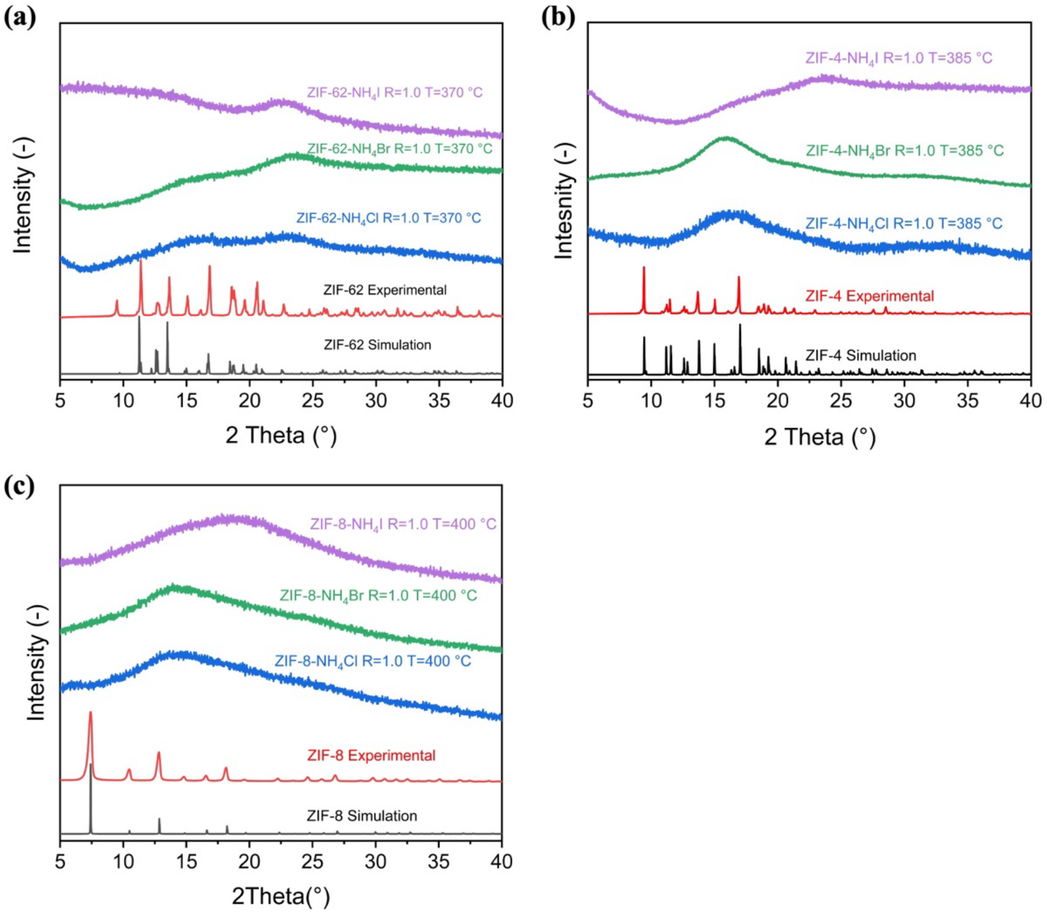

Supplementary Figure 2. XRD patterns of **(a)** ZIF-62, **(b)** ZIF-4, and **(c)** ZIF-8 crystals and melt-quenched with different halide salts at R=1 and heated to varying maximum temperatures (370-400 °C).

Supplementary Figure 3

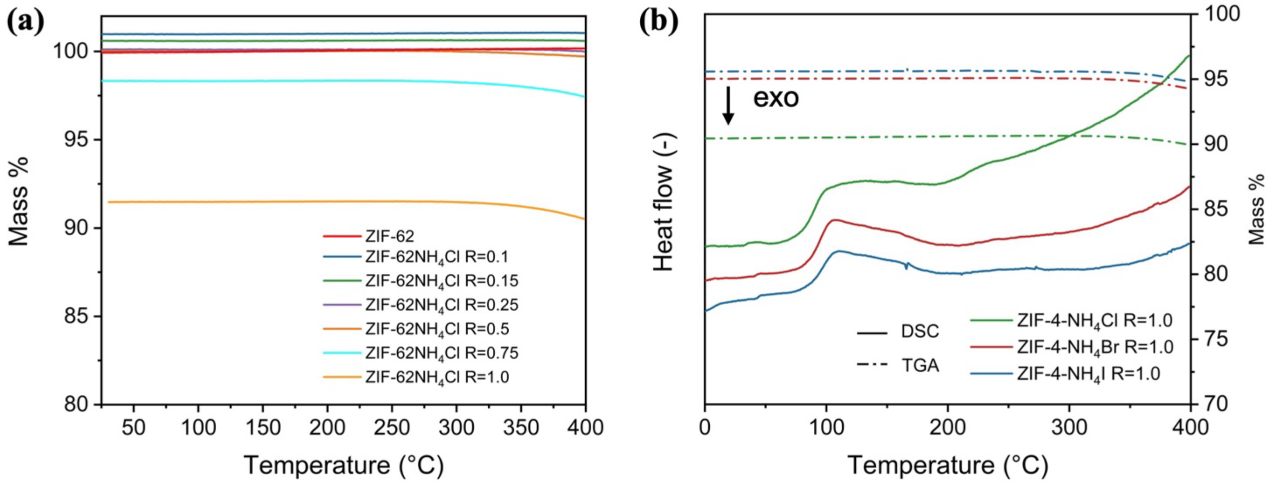

Supplementary Figure 3. (a) TGA heating trace for ZIF-62-NH_4Cl with different *R* values (from 0 to 1.0). (b) DSC and TGA heating traces for ZIF-4-NH_4X, [X=Cl, Br, and I] with *R* = 1.0. Data in both panels have been collected at rate of 10 K min^{-1} in PtRh crucible. Heat flow data have been shifted vertically for clarity. All curves are the second upscan of each sample after an initial upscan and downscan to reset the thermal history of the glass (causing the TGA curves to not initiate at 100%).

Supplementary Figure 4

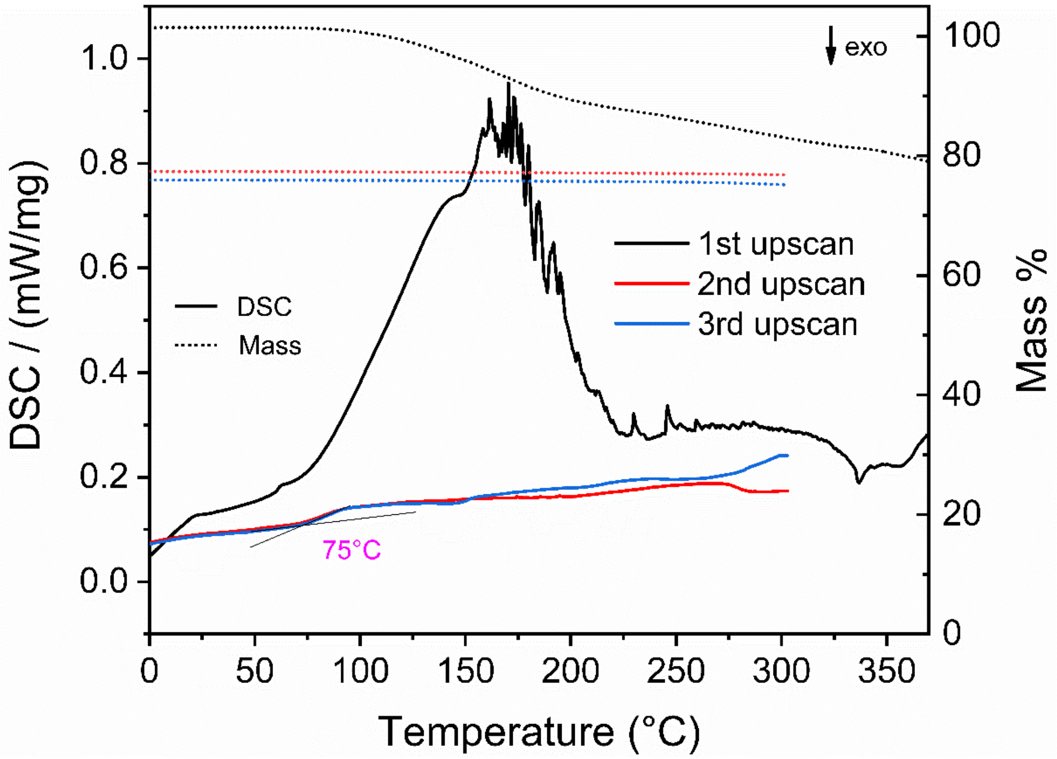

Supplementary Figure 4. Three consecutive DSC and TGA heating scans (at 10 K min^{-1}, same cooling rate) of the ZIF-62 crystal mixed with NH_4Cl for R=1.0. The broad endothermic feature observed during the first heating cycle likely represents melting as well as release of gaseous species, which is accompanied by a detectable mass loss. The second and third heating scans display a sharp and reproducible transition at the same temperature range, with no associated mass loss, indicating a typical glass transition behavior of the hybrid glass obtained after the first thermal cycle.

Supplementary Figure 5

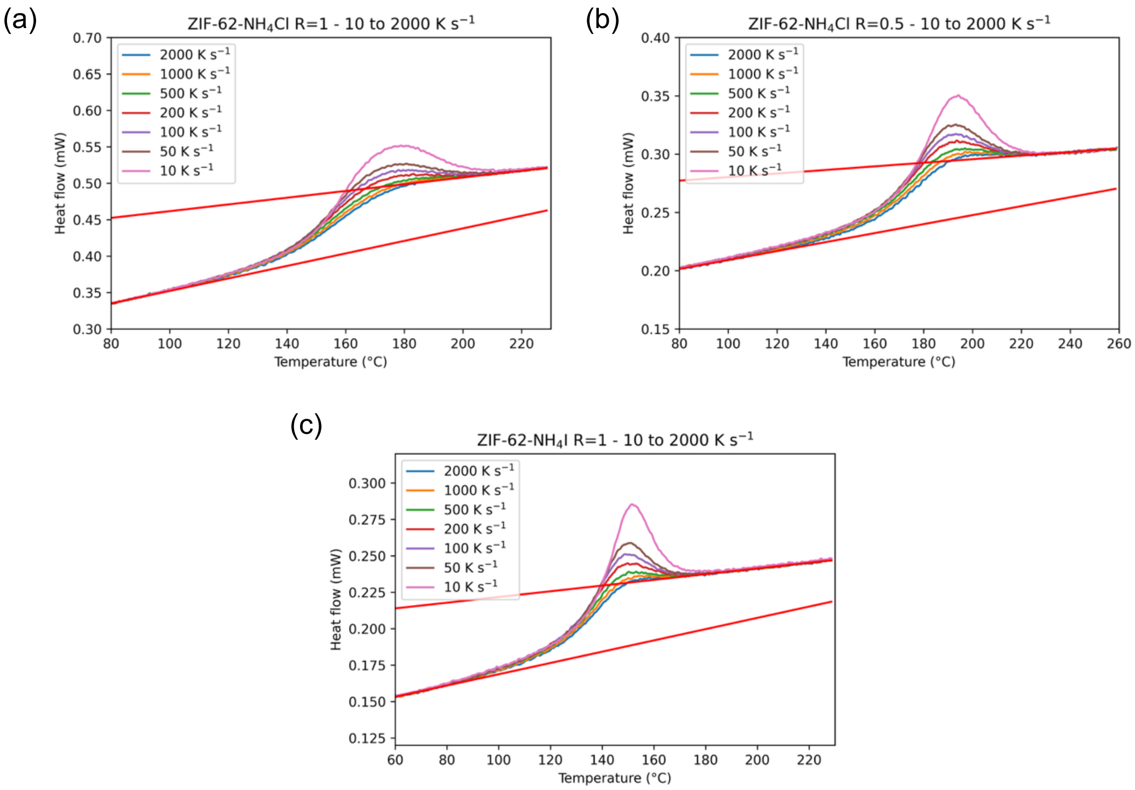

Supplementary Figure 5. FDSC scans for a constant heating rate of 1000 K s^{-1} but with preceding cooling rates ranging from 10 to 2000 K s^{-1}. Results are shown for (a) ZIF-62-NH_4Cl *R*=1.0, (b) ZIF-62-NH_4Cl *R*=0.5, and (c) ZIF-62-NH_4I *R*=1.0 samples. The red lines mark the extrapolation of the heat flow, corresponding to the heat capacity of the glass and liquid states.

Supplementary Figure 6

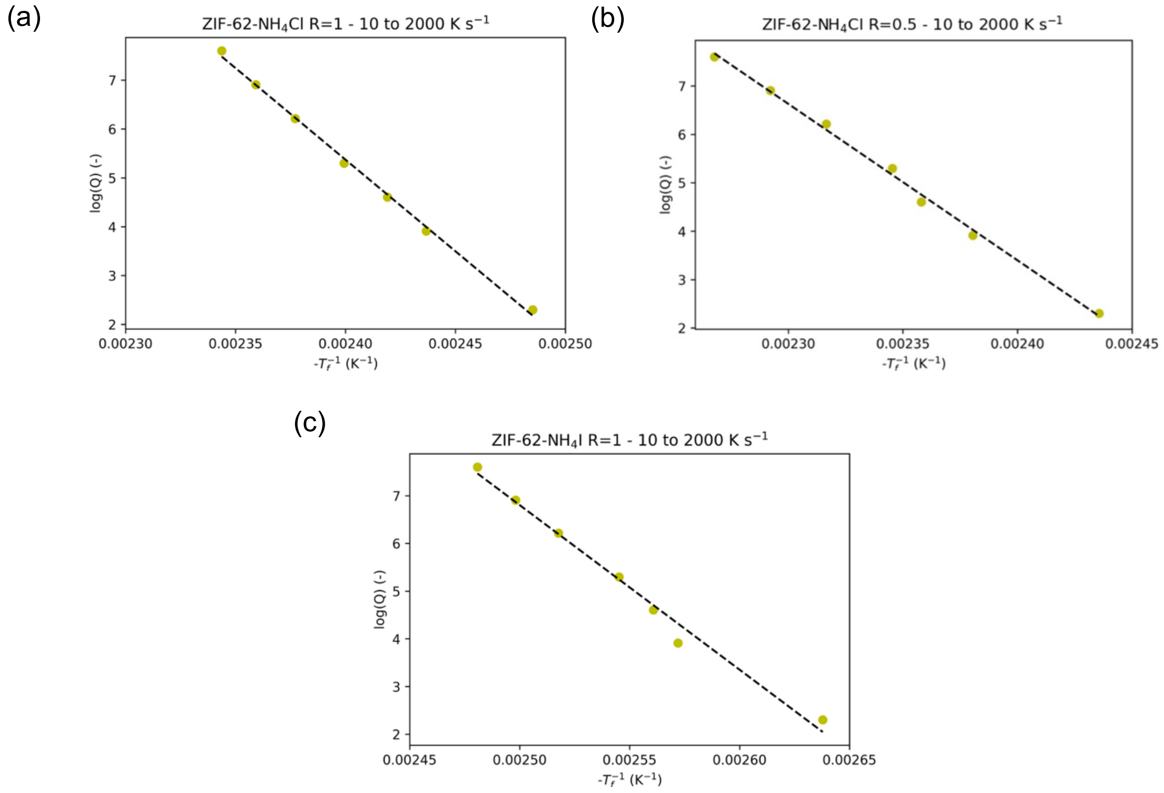

Supplementary Figure 6. Relation between cooling rate (Q_c) and inverse of the fictive temperature (T_f) for (a) ZIF-62-NH_4Cl R=1.0, (b) ZIF-62-NH_4Cl R=0.5, and (c) ZIF-62-NH_4I R=1.0 samples, as determined from the FDSC measurements. The slope was used to estimate the activation energy for the onset of the glass transition (see Methods section for details).

Supplementary Figure 7

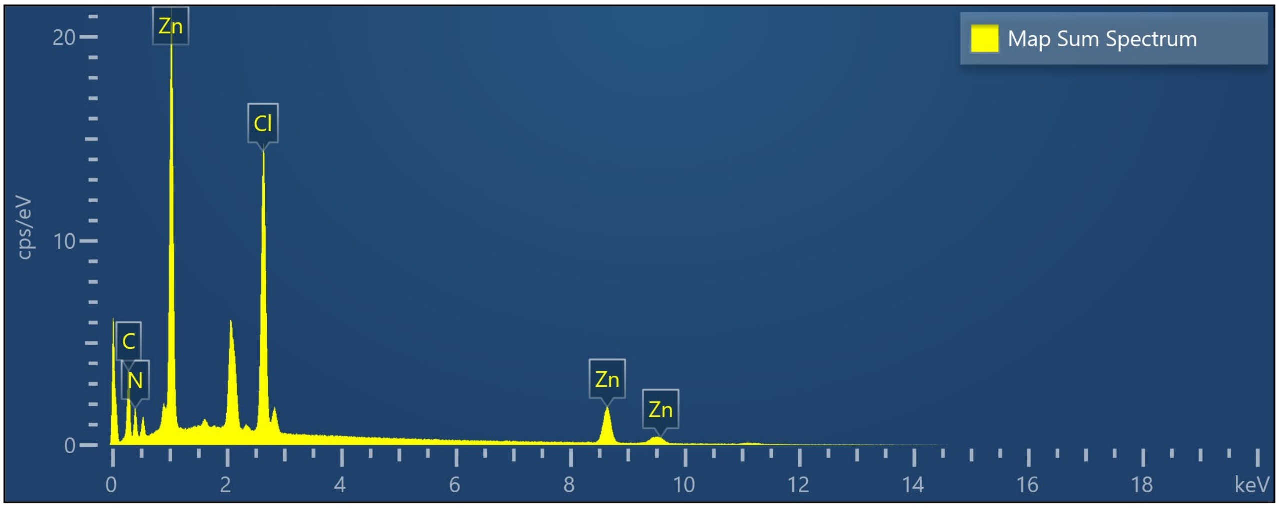

Supplementary Figure 7. Energy-dispersive X-ray spectroscopy spectrum of the ZIF-62-NH_4Cl glass (*R*=1.0), confirming the presence of C, N, Cl, and Zn elements.

Supplementary Figure 8

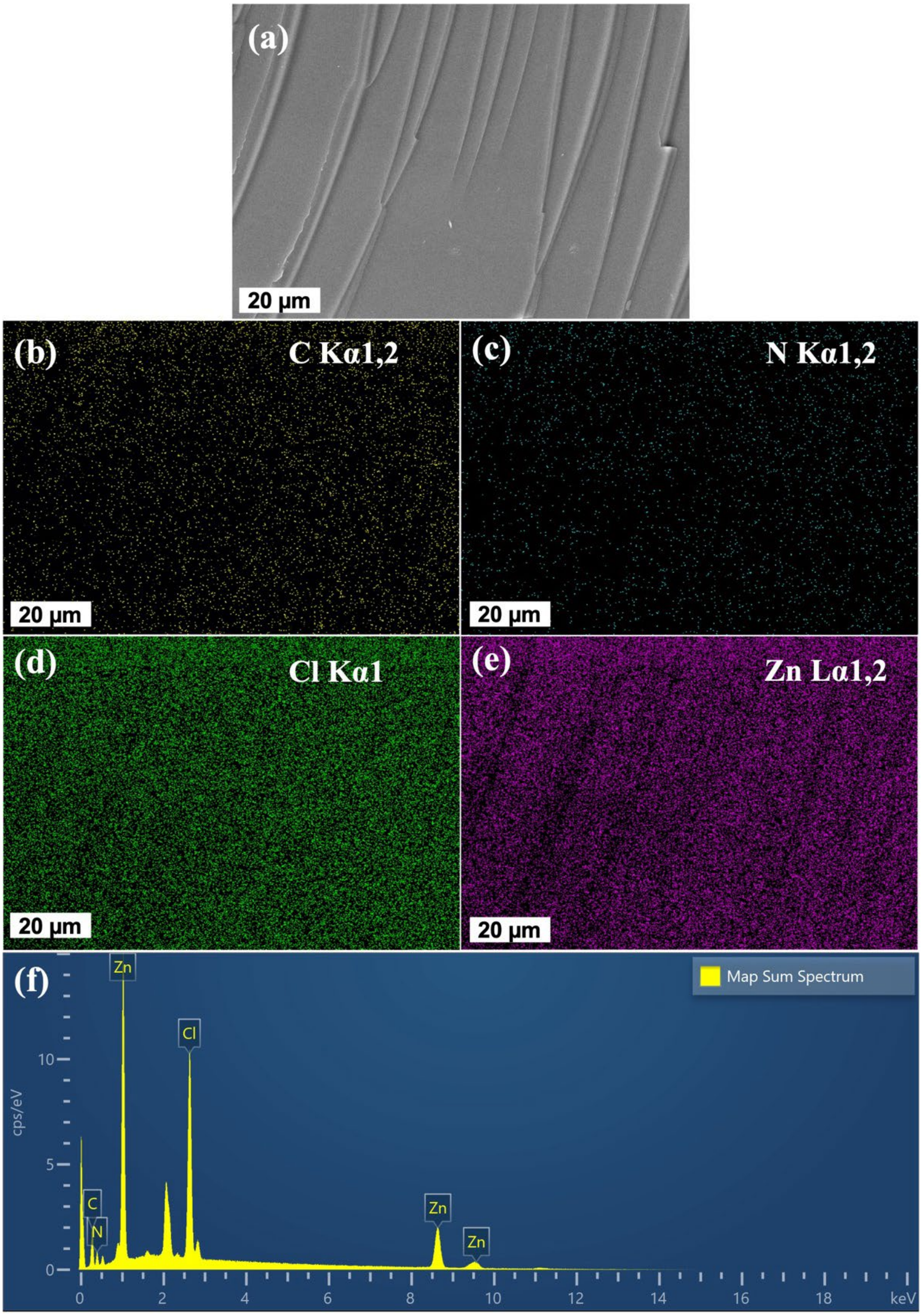

Supplementary Figure 8. SEM-EDX elemental mapping of the ZIF-62-NH_4Cl glass for R = 0.5. (a) SEM image of the sample surface. (b-e) Elemental mapping showing the spatial distribution of (b) C, (c) N, (d) Cl, and (e) Zn elements. (f) EDX spectrum showing the presence of C, N, Cl, and Zn elements.

Supplementary Figure 9

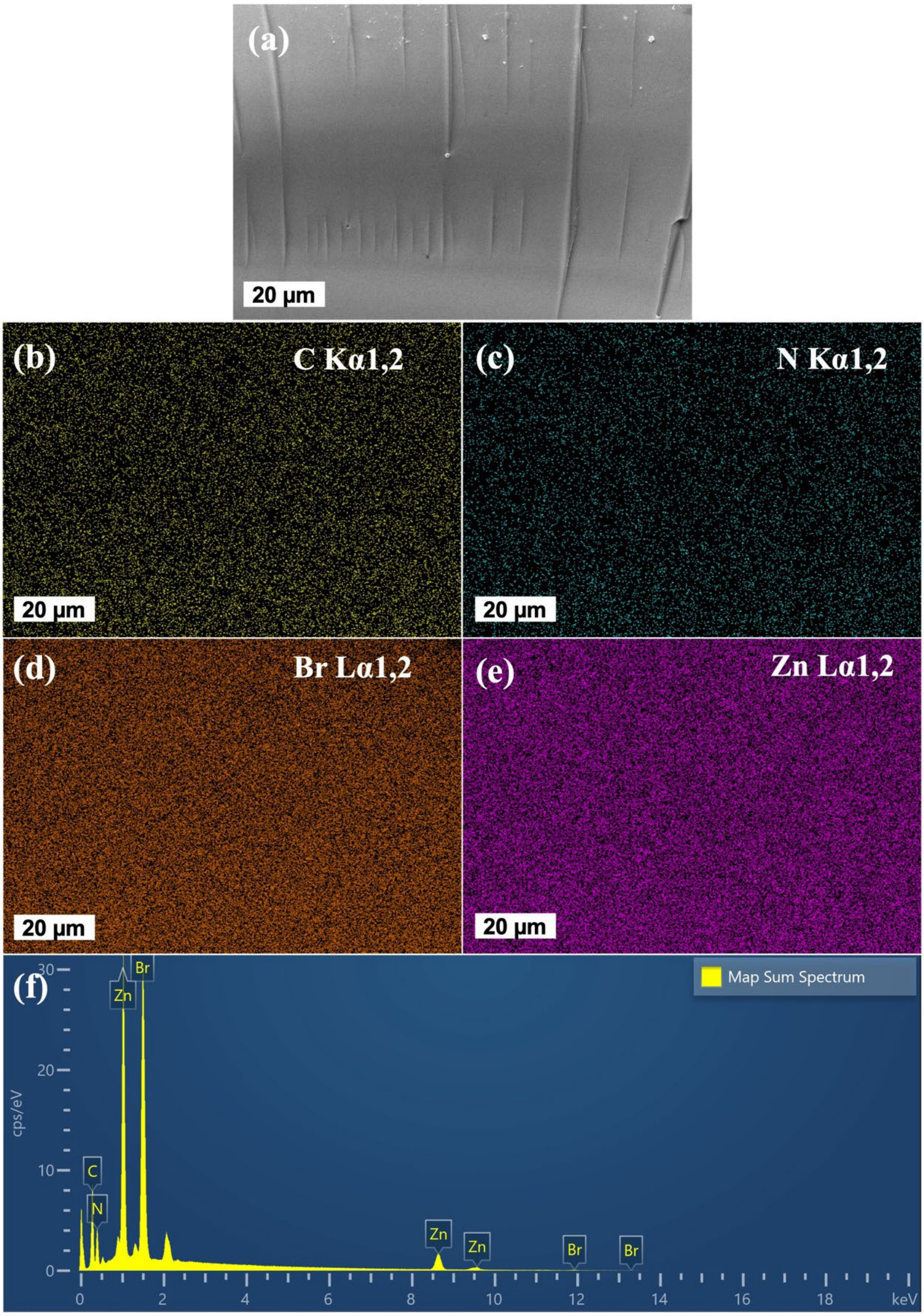

Supplementary Figure 9. SEM-EDX elemental mapping of the ZIF-62-NH_4Br glass for R=1.0. (a) SEM image of the sample surface. (b-e) Elemental mapping showing the spatial distribution of (b) C, (c) N, (d) Br, and (e) Zn. (f) EDX spectrum showing the presence of C, N, Br, and Zn elements.

Supplementary Figure 10

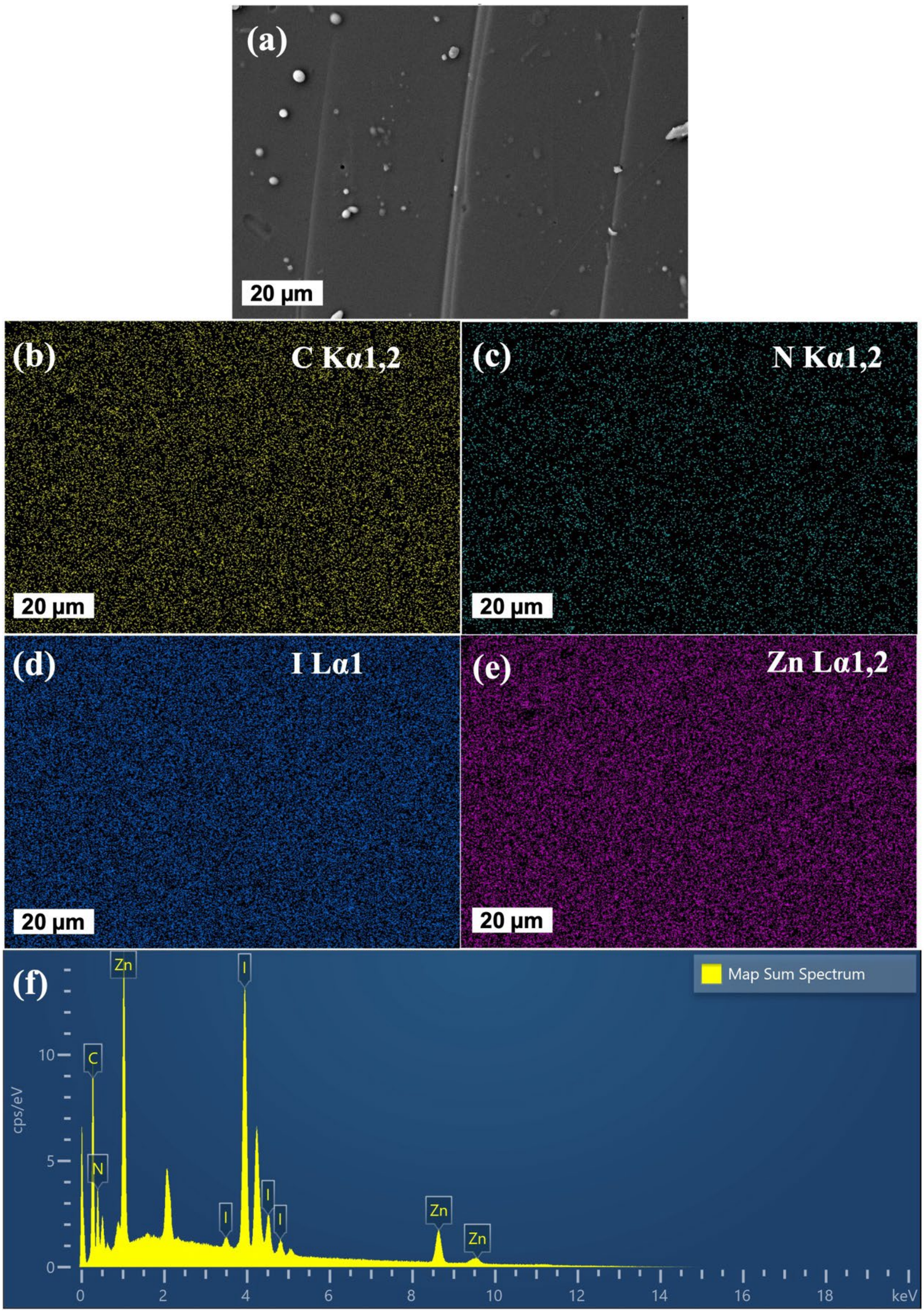

Supplementary Figure 10. SEM-EDX elemental mapping of the ZIF-62-NH_4I glass for R=1.0. (a) SEM image of the sample surface. (b-e) Elemental mapping showing the spatial distribution of (b) C, (c) N, (d) I, and (e) Zn. (f) EDX spectrum showing the presence of C, N, I, and Zn elements.

Supplementary Figure 11

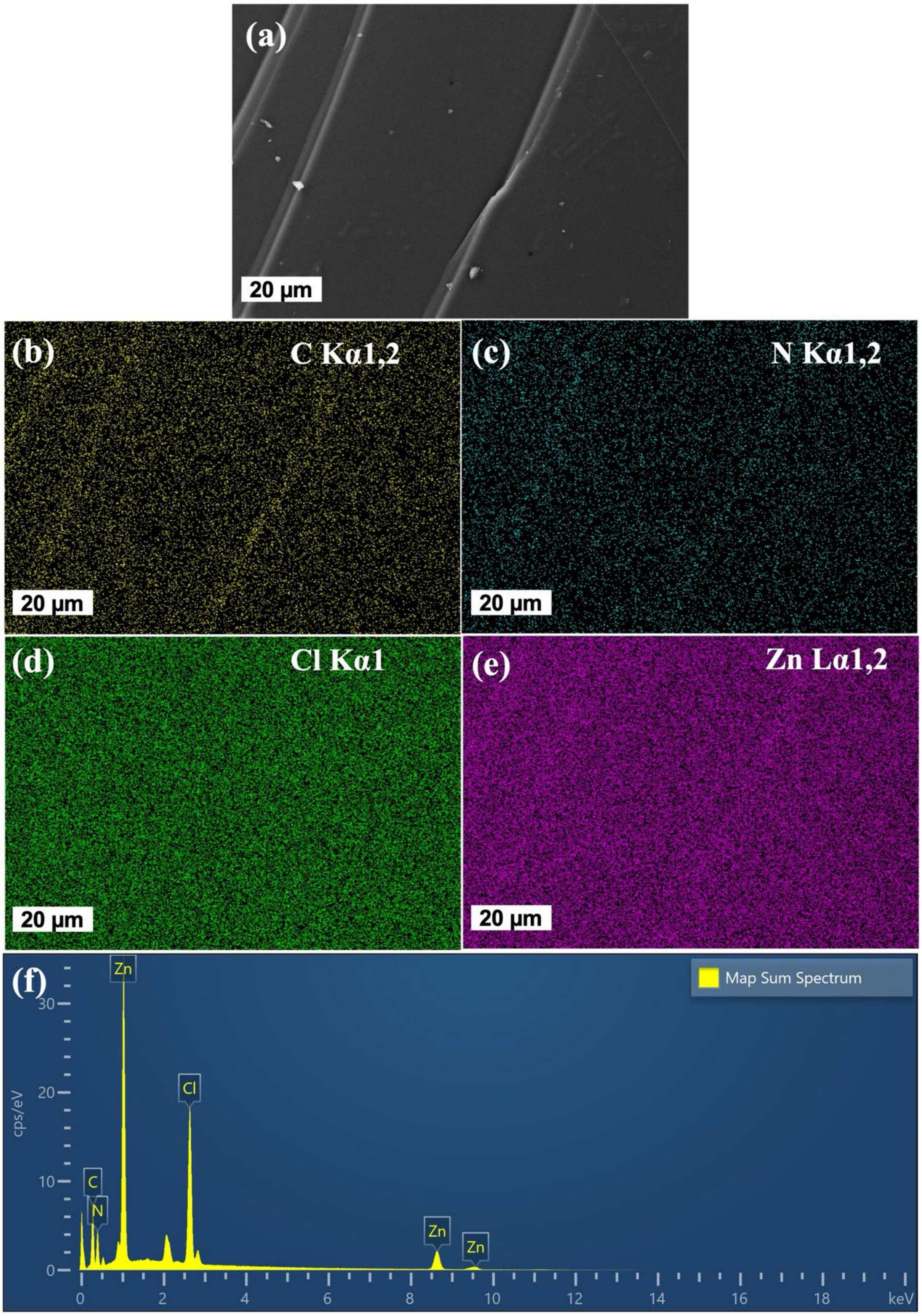

Supplementary Figure 11. SEM-EDX elemental mapping of the ZIF-4-NH_4Cl glass for R=1.0. (a) SEM image of the sample surface. (b-e) Elemental mapping showing the spatial distribution of (b) C, (c) N, (d) Cl, and (e) Zn. (f) EDX spectrum showing the presence of C, N, Cl, and Zn elements.

Supplementary Figure 12

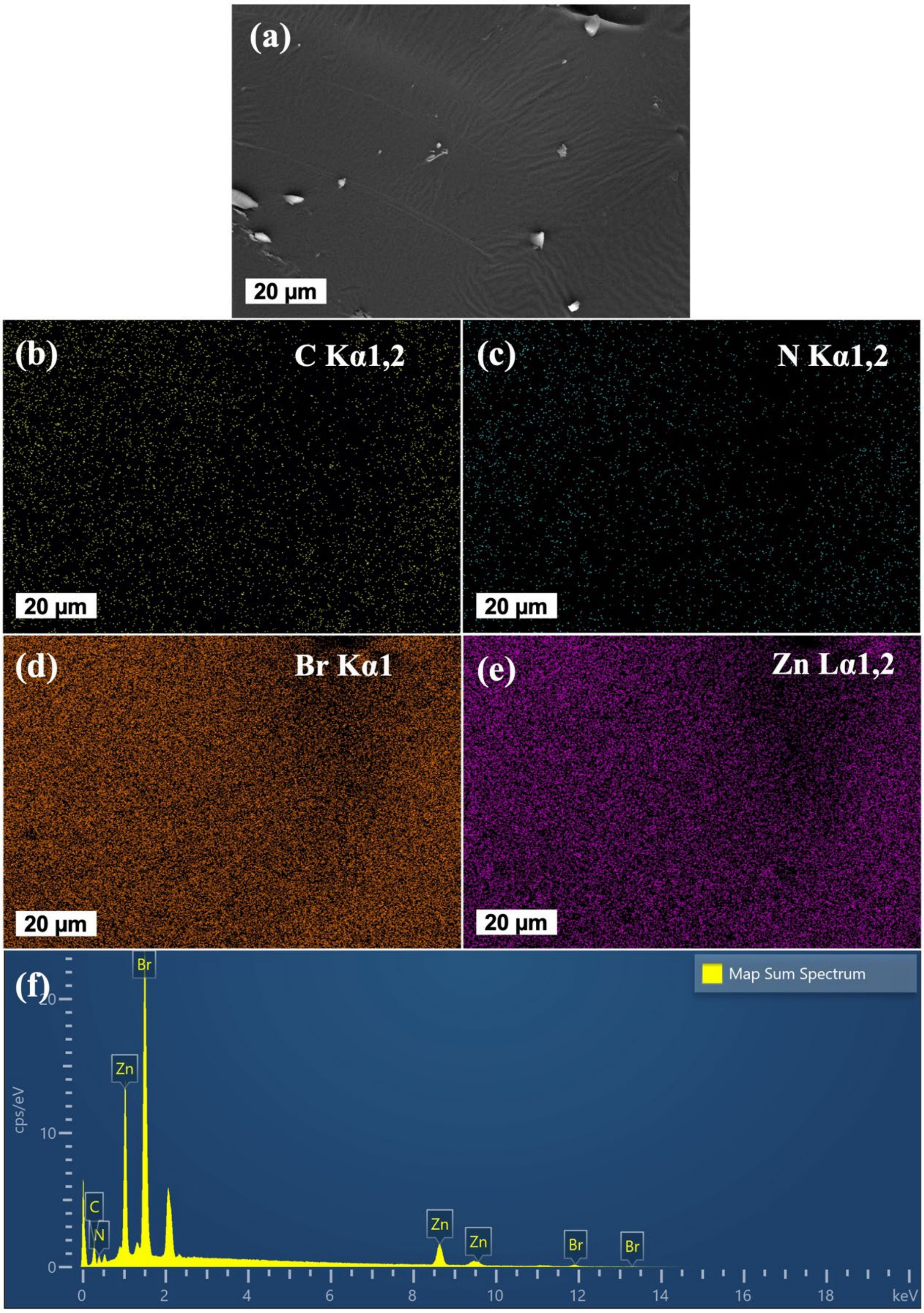

Supplementary Figure 12. SEM-EDX elemental mapping of the ZIF-8-NH_4Br glass for R=1.0. (a) SEM image of the sample surface. (b-e) Elemental mapping showing the spatial distribution of (b) C, (c) N, (d) Br, and (e) Zn. (f) EDX spectrum showing the presence of C, N, Cl, and Zn elements.

Supplementary Figure 13

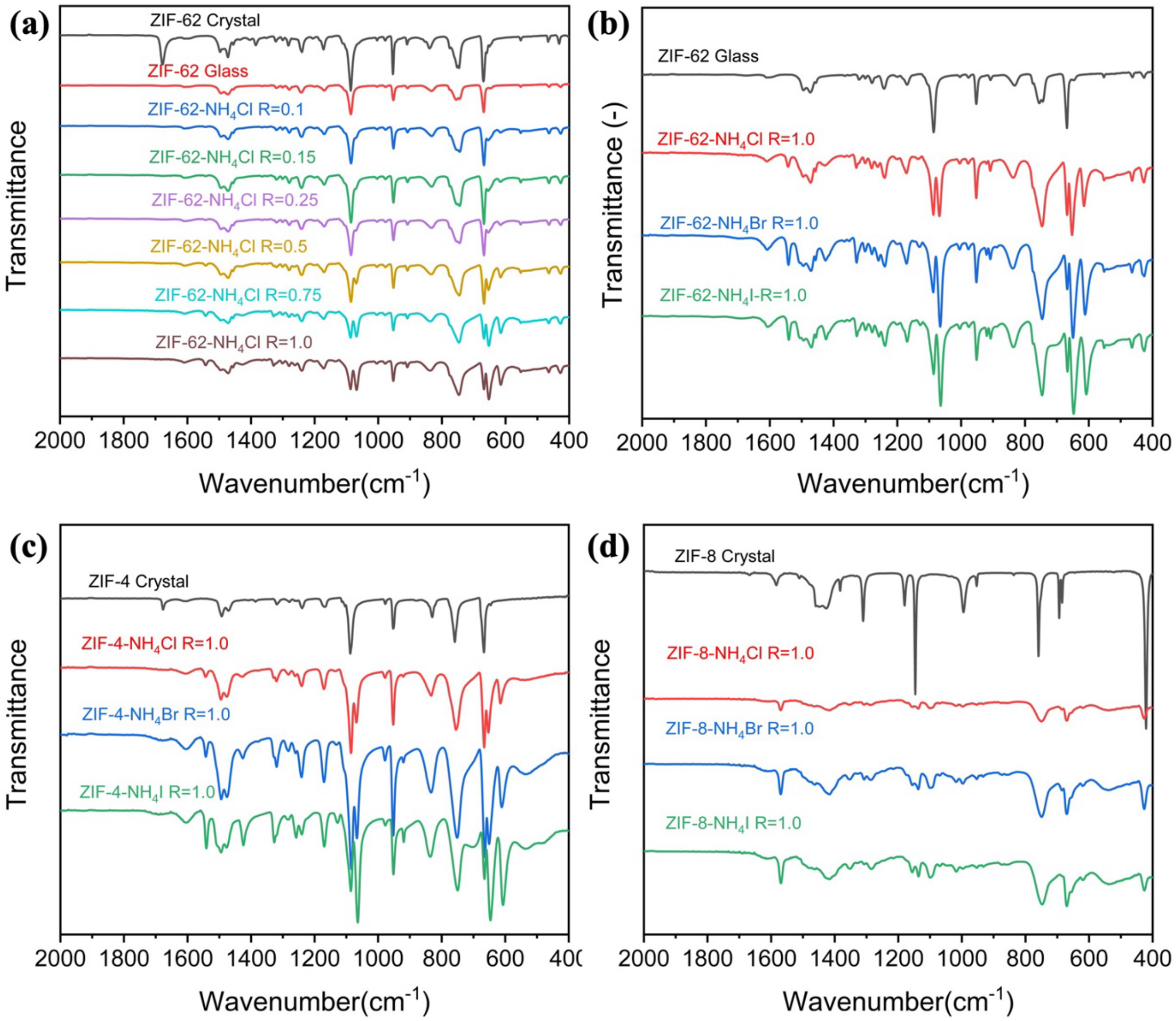

Supplementary Figure 13. Fourier transform infrared transmission spectra of modified ZIF-derived glasses with varying *R* values for different ammonium salts. (a) ZIF-62 modified with NH_4Cl at different *R* values (*R* of 0.1 to 1.0). (b) ZIF-62 modified with different NH_4X salts (X = Cl, Br, I). (c) ZIF-4 modified with different NH_4X salts. (d) ZIF-8 modified with different NH_4X salts.

Supplementary Figure 14

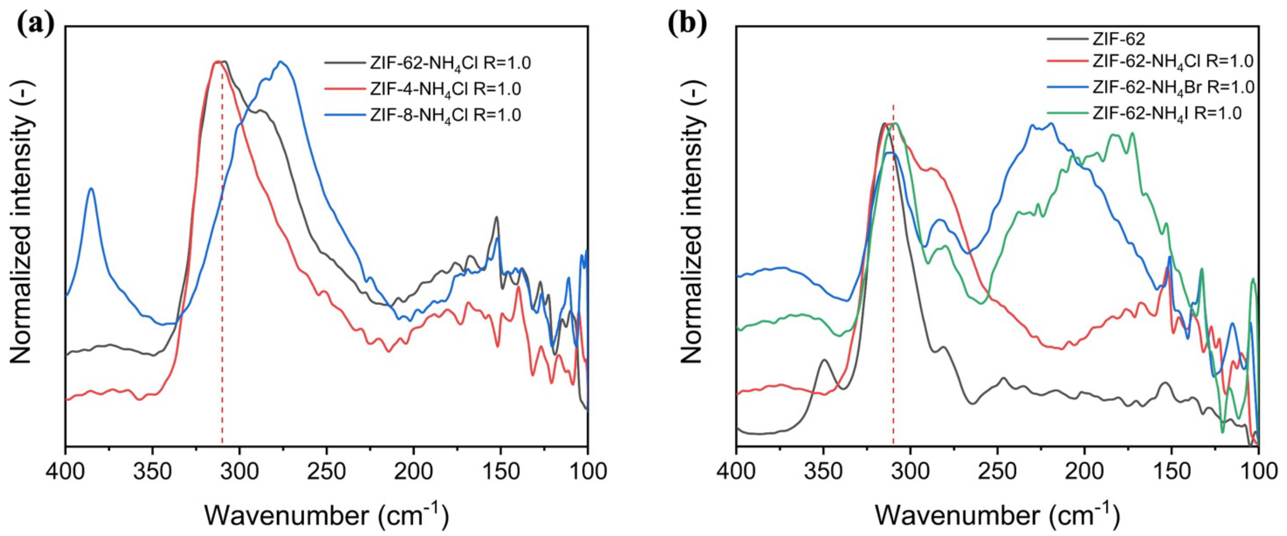

Supplementary Figure 14. Far-infrared reflectance spectra of (a) ZIF-62/4/8-NH_4Cl glasses with constant R=1.0, and (b) ZIF-62-NH_4X glasses with constant R=1.0 for X = Cl, Br, and I.

Supplementary Figure 15

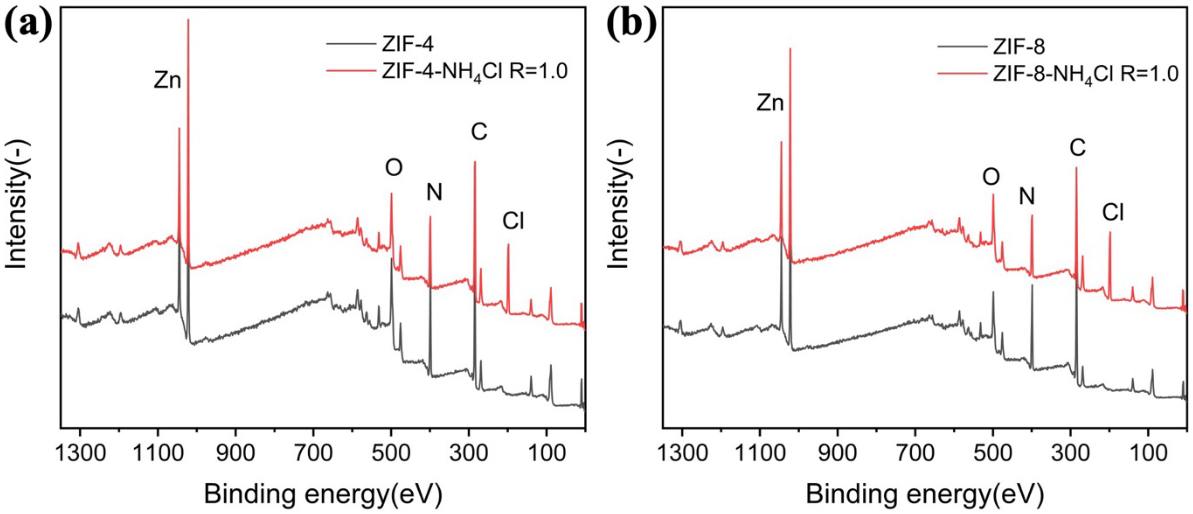

Supplementary Figure 15. X-ray photoelectron spectroscopy data of the ZIF crystals as well as modified ZIF glasses. (a) ZIF-4 crystal and ZIF-4-NH_4Cl glass at R=1.0. (b) ZIF-8 crystal and ZIF-8-NH_4Cl glass at R=1.0.

Supplementary Figure 16

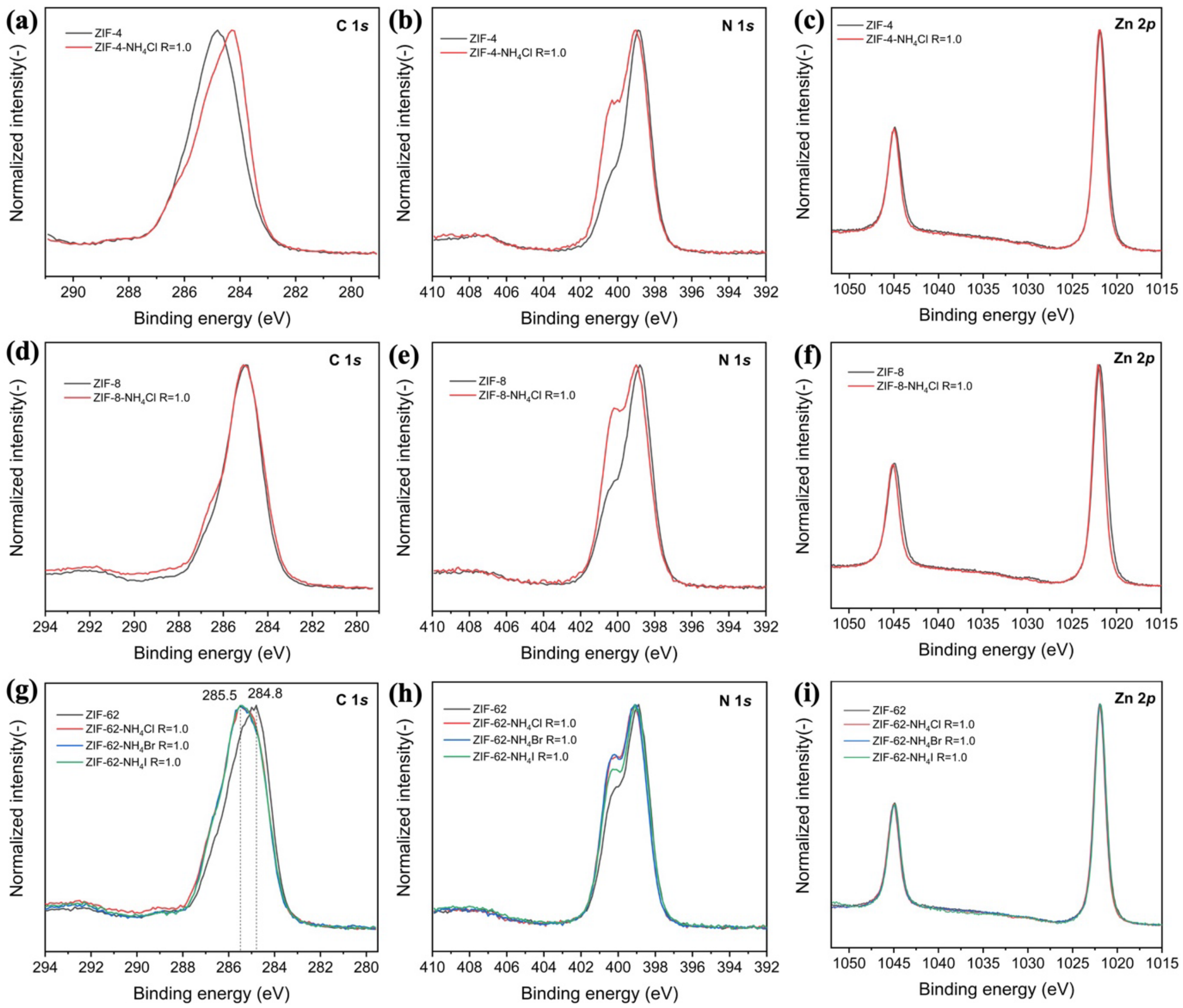

Supplementary Figure 16. (a-c) X-ray photoelectron spectroscopy (XPS) data for (a) C 1s, (b) N 1s, and (c) Zn 2p of ZIF-4 and ZIF-4-NH_4Cl *R*=1.0 glasses. (d-f) XPS data for (d) C 1s, (e) N 1s, and (f) Zn 2p of ZIF-8 and ZIF-8-NH_4Cl R=1.0 glasses. (g-i) XPS data for (g) C 1s, (h) N 1s, and (i) Zn 2p of ZIF-62 and ZIF-62-NH_4X *R*=1.0 glasses (X=Cl, Br, and I).

Supplementary Figure 17

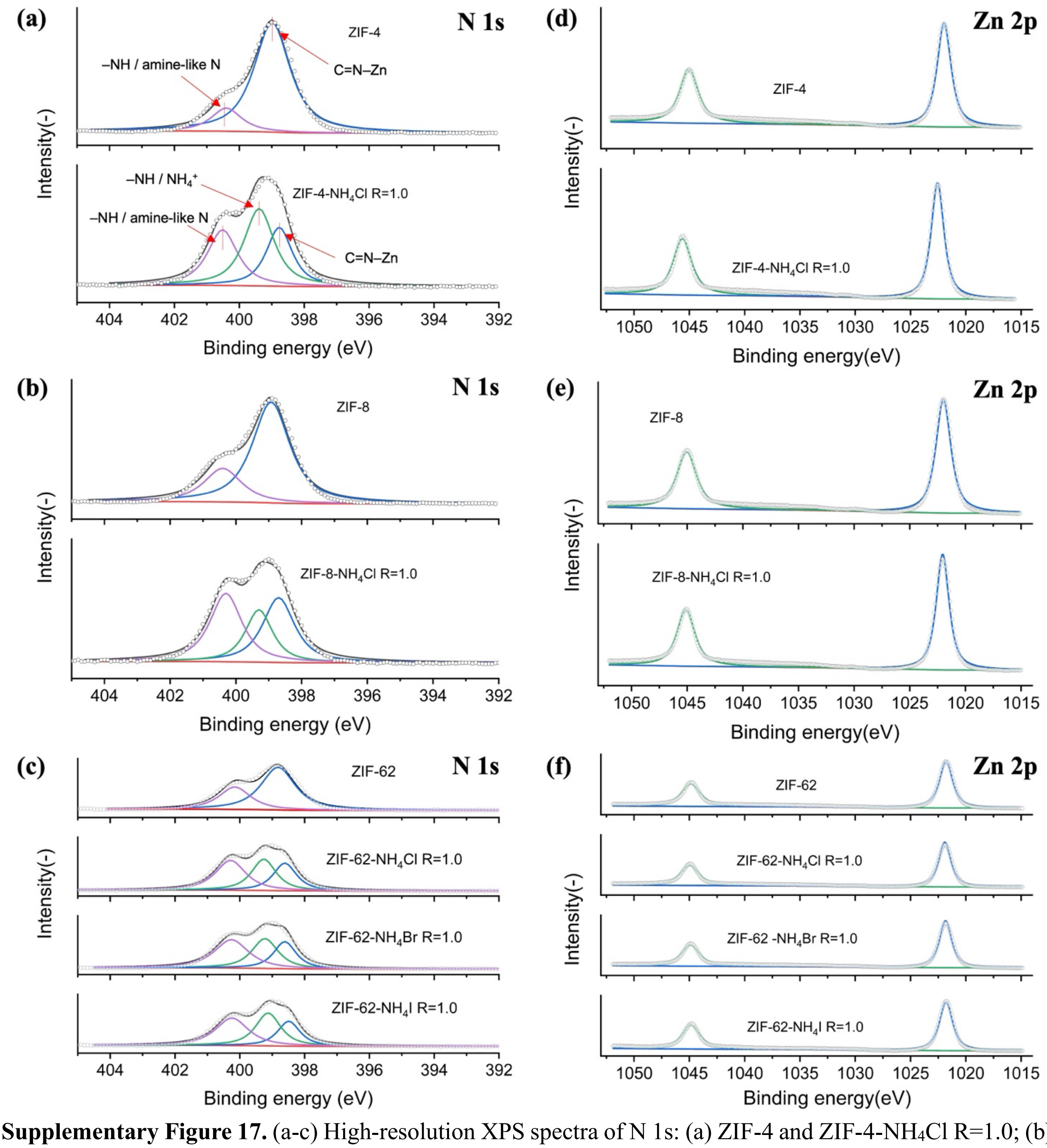

Supplementary Figure 17. (a-c) High-resolution XPS spectra of N 1s: (a) ZIF-4 and ZIF-4-NH_4Cl R=1.0; (b) ZIF-8 and ZIF-8-NH_4Cl R=1.0; and (c) ZIF-62, ZIF-62-NH_4Cl R=1.0, ZIF-62-NH_4Br R=1.0, ZIF-62-NH_4I R=1.0. (d-f) High-resolution XPS spectra of Zn 2p: (d) ZIF-4 and ZIF-4-NH_4Cl R=1.0; (e) ZIF-8 and ZIF-8-NH_4Cl R=1.0; and (f) ZIF-62, ZIF-62-NH_4Cl R=1.0, ZIF-62-NH_4Br R=1.0, ZIF-62-NH_4I R=1.0.

Supplementary Figure 18

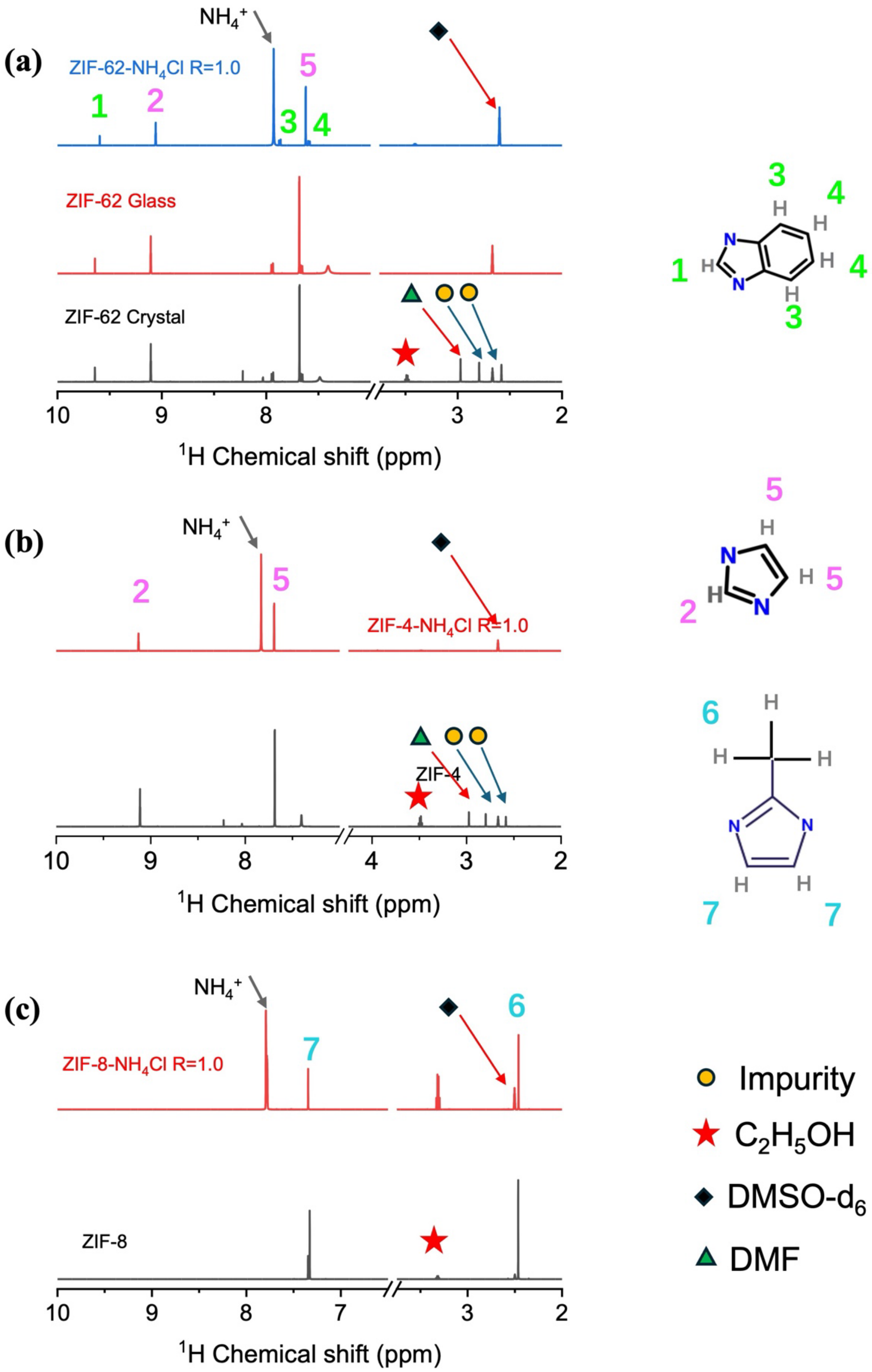

Supplementary Figure 18. Liquid state ^{1}H NMR spectra of (a) ZIF-62 crystal, ZIF-62 glass, and ZIF-62-NH_4Cl *R*=1.0, (b) ZIF-4 crystal and ZIF-4-NH_4Cl *R*=1.0, and (c) ZIF-8 crystal and ZIF-8-NH_4Cl *R*=1.0. All samples were probed in DMSO-d_6.

Supplementary Figure 19

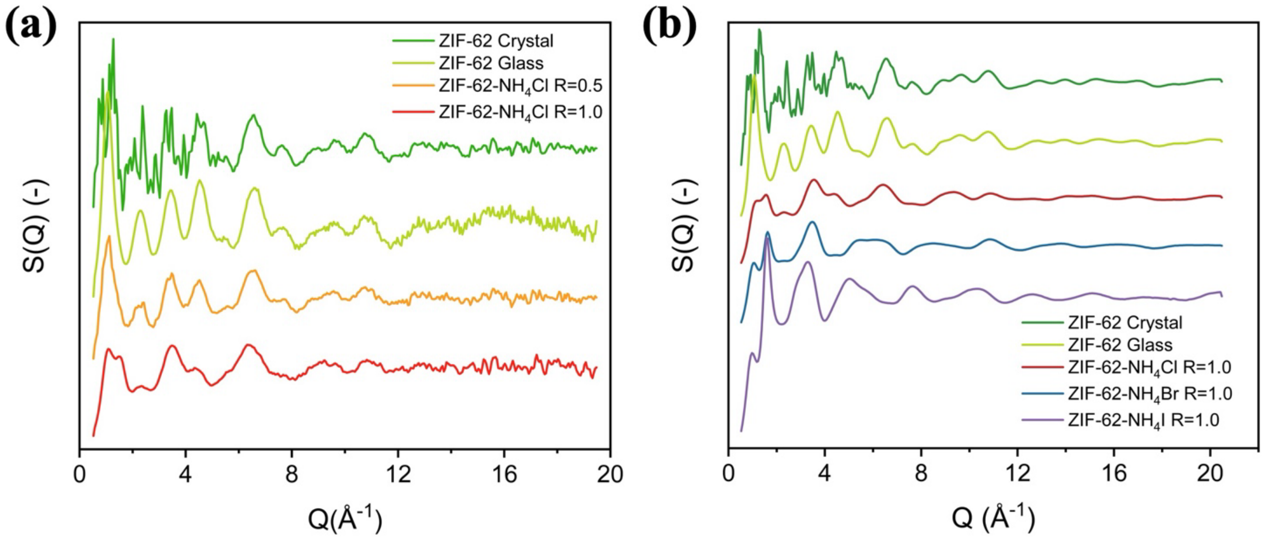

Supplementary Figure 19. X-ray structure factor $S(Q)$. (a) ZIF-62 crystal and glass, and ZIF-62-NH_4Cl glass with R of 0.5 and 1.0. (b) ZIF-62 crystal and glass, and ZIF-62-NH_4Cl, ZIF-62-NH_4Br and ZIF-62-NH_4I glasses with R=1.0.

Supplementary Figure 20

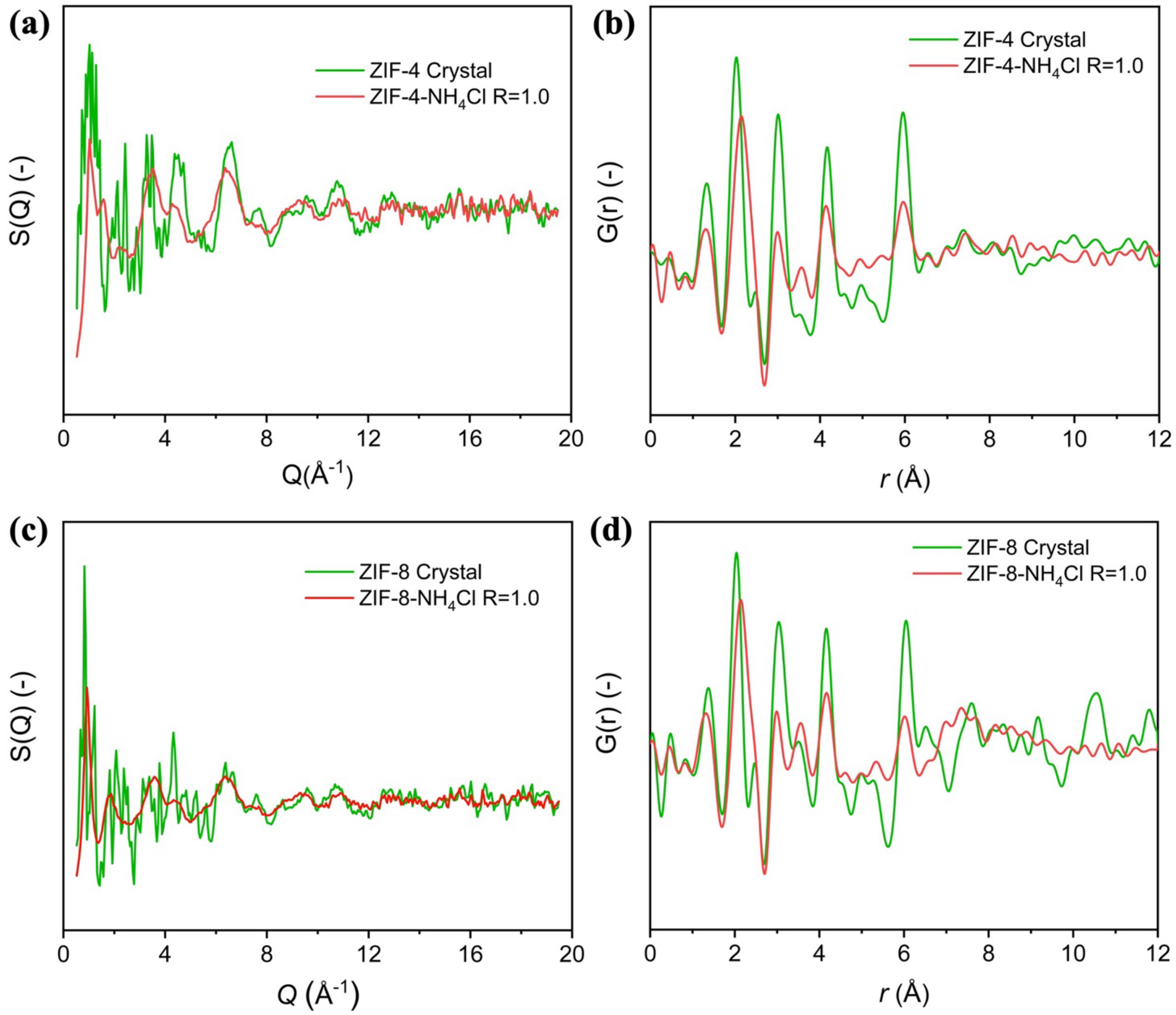

Supplementary Figure 20. X-ray structure factor *S*(*Q*) and pair-distribution function *G*(*r*) data. (a) *S*(*Q*) and (b) *G*(*r*) for ZIF-4 crystal and ZIF-4-NH_4Cl glass with *R*=1.0. (c) *S*(*Q*) and (d) *G*(*r*) for ZIF-8 crystal and ZIF-8-NH_4Cl glass with *R*=1.0.

Supplementary Figure 21

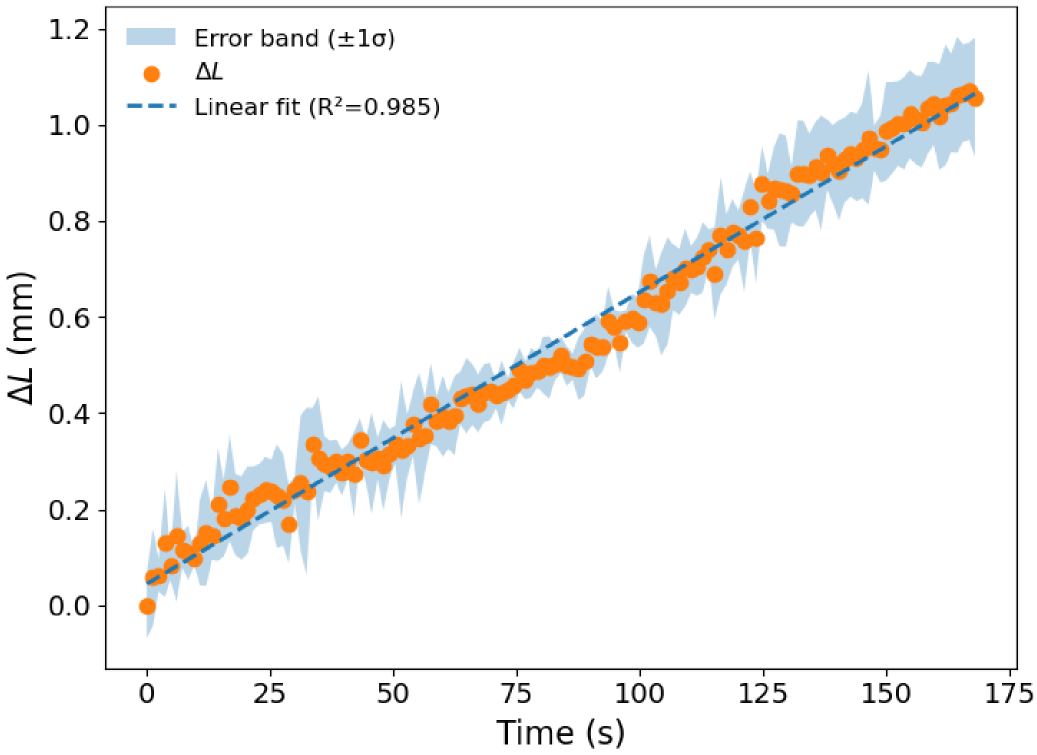

Supplementary Figure 21. Evolution of the interfacial reaction layer thickness (ΔL) between ZIF-62 and NH_4Cl during *in situ* X-ray imaging experiments. The interfacial growth was quantified from 311 s to 479 s in Supplementary Video 1, corresponding to a temperature increase from 286 to 343 °C. A linear fit of the interfacial thickness as a function of time yields $\Delta L = 0.00607t + 0.0451$ with a coefficient of determination of R^2=0.985.
